# *De novo* genome assembly of the partial homozygous dihaploid potato identified PVY resistance gene (*Ry_chc_*) derived from *Solanum chacoense*

**DOI:** 10.1270/jsbbs.22078

**Published:** 2023-04-13

**Authors:** Kotaro Akai, Kenji Asano, Chika Suzuki, Etsuo Shimosaka, Seiji Tamiya, Takako Suzuki, Toru Takeuchi, Takehiro Ohki

**Affiliations:** 1 Memuro Upland Farming Research Division, Hokkaido Agricultural Research Center, National Agricultural Research Organization, Memuro, Hokkaido 082-0081, Japan; 2 Research Faculty of Agriculture, Hokkaido University, Sapporo, Hokkaido 060-8589, Japan; 3 Hokkaido Research Organization, Central Agricultural Experiment Station, Naganuma, Hokkaido 069-1395, Japan; 7 National Agriculture and Food Research Organization, Hokkaido Agricultural Research Center, Sapporo, Hokkaido 062-8555, Japan

**Keywords:** gene isolation, resistance gene, *Ry_chc_*, polyploidy, *de novo* assembly, third-generation sequencing

## Abstract

The isolation of disease resistance genes introduced from wild or related cultivated species is essential for understanding their mechanisms, spectrum and risk of breakdown. To identify target genes not included in reference genomes, genomic sequences with the target locus must be reconstructed. However, *de novo* assembly approaches of the entire genome, such as those used for constructing reference genomes, are complicated in higher plants. Moreover, in the autotetraploid potato, the heterozygous regions and repetitive structures located around disease resistance gene clusters fragment the genomes into short contigs, making it challenging to identify resistance genes. In this study, we report that a *de novo* assembly approach of a target gene-specific homozygous dihaploid developed through haploid induction was suitable for gene isolation in potatoes using the potato virus Y resistance gene *Ry_chc_* as a model. The assembled contig containing *Ry_chc_*-linked markers was 3.3 Mb in length and could be joined with gene location information from the fine mapping analysis. *Ry_chc_* was successfully identified in a repeated island located on the distal end of the long arm of chromosome 9 as a Toll/interleukin-1 receptor-nucleotide-binding site-leucine rich repeat (TIR-NBS-LRR) type resistance gene. This approach will be practical for other gene isolation projects in potatoes.

## Introduction

The isolation of the causal genes responsible for disease resistance in crops is not only an essential study for elucidating the disease resistance mechanism but is also important for evaluating its utility in breeding, such as estimating resistance intensity and spectrum and the risk of resistance breakdown. In potato (*Solanum tuberosum* L.) breeding programs, many resistance genes against late blight (Paluchowska *et al.* 2022), potato cyst nematode (Gartner *et al.* 2021), potato virus Y (PVY) (Grech-Baran *et al.* 2020, Nie *et al.* 2016, Szajko *et al.* 2014), potato virus X (PVX) (Ritter *et al.* 1991, Van der Vossen *et al.* 2000), bacterial wilt caused by *Ralstonia solanacearum* (Habe *et al.* 2019, Mori *et al.* 2012) have been introduced, but some of resistance genes are still not cloned.

In recent years, several methods used to obtain high-density genotypes linked to phenotypes have become more accessible by combining high-quality reference genomes with next-generation sequencing (NGS)-based techniques, such as double-digest restriction site-associated DNA sequencing (ddRAD-seq) (Shirasawa *et al.* 2016), Genotyping by random amplicon sequencing-direct (GRAS-Di) (Enoki and Takeuchi 2018, Hosoya *et al.* 2019), MutMap+ (Fekih *et al.* 2013), and QTL-seq (Takagi *et al.* 2013, Yamakawa *et al.* 2021). These technologies have enabled us to obtain the locus positions of target genes and information on linked DNA markers for fine mapping at high speed and with high accuracy. However, regardless of how much candidate regions are narrowed down by fine mapping, isolating disease resistance genes derived from wild or cultivated relatives that are distantly related to the reference genome remains challenging. This is because the reference genomes do not contain these genes, the resistance genes are often clustered, and the region involves structural variations associated with gene duplications. In such cases, the sequence of the target gene locus must be reconstructed newly.

There are two significant issues in the isolation of target genes via a *de novo* assembly approach in polyploid crops. First, genome assemblers cannot reconstruct the heterozygous regions into one contiguous sequence because the assemblers fragment contigs to preserve information on haplotypes (Garg *et al.* 2018, Kajitani *et al.* 2014, 2019, Schatz *et al.* 2012). To avoid heterozygosity, most of the previously reported reference genomes of potatoes were reconstructed from pure lines or doubled monoploids (Pham *et al.* 2020, Van Lieshout *et al.* 2020, Xu *et al.* 2011). Second, the repetitive structure caused by gene duplication events or translocation events of transposable elements also causes fragmentation of contigs (Alkan *et al.* 2011, Schatz *et al.* 2012). For these reasons, if the same approach as that used in the previous reference genome construction projects was selected for gene isolation, the cost of creating materials, sequencing, and computer analysis would be high. Particularly, the autotetraploid potato has highly heterozygous genomes; therefore, it is nearly impossible to assemble contigs that are sufficiently long for map-based cloning.

However, when information about the target gene locus is available, chromosome-scale genome assembly is unnecessary for gene isolation (Grech-Baran *et al.* 2020). If we can create lines homozygous in the target region, we should be able to obtain the sequence of the target locus with high accuracy using the *de novo* genome assembly approach. In the case of potatoes, homozygous dihaploid lines (RR or rr) could be obtained from dihaploid induction (Busse *et al.* 2021, Hutten *et al.* 1993, Peloquin *et al.* 1996, Van Breukelen *et al.* 1977) of duplex tetraploid lines (RRrr) with a probability of 1/6. On the other hand, homozygous tetraploid lines (RRRR or rrrr) could be obtained with a probability of 1/36 from crosses of duplex tetraploid RRrr × RRrr. Therefore, it is easier to obtain the homozygous line by the dihaploid induction approach than crossing in the tetraploid level. Also, in the allele dosage estimation analysis by quantitive PCR or progeny tests, homozygosity is easier to determine in dihaploid than in tetraploids. Such target gene specific homozygous dihaploid lines would be unsuitable for *de novo* assembly of the entire genome because most of the genome is heterozygous, but they are helpful for the assembly aimed at targeted gene isolation.

Moreover, third-generation sequencing (TGS) is effective in solving repetitive structures. The nanopore sequencing technology offered by Oxford Nanopore Technology and the single molecule real-time (SMRT) sequencing technology offered by Pacific Biosciences can output very long reads (>15 kb). These long reads can overcome the scattered repeats in the genome, thus facilitating the resolution of graph structures during *de novo* assembly and significantly improving the continuity of contigs (Kono and Arakawa 2019).

*Ry_chc_* derived from an wild potato (*S. chacoense*) is a valuable gene for potato breeding programs, as it confers extreme resistance against PVY infection. *Ry_chc_* was first introduced into the commercial cultivar ‘Konafubuki’ from doubled *S. chacoense* ‘w84’(Asama *et al.* 1982) and was mapped to the distal end of the long arm of chromosome 9 (Hosaka *et al.* 2001, Sato *et al.* 2006, Takeuchi *et al.* 2008). Moreover, Li *et al.* (2022) reported the cloning of the *Ry_chc_* gene as a Toll/interleukin-1 receptor (TIR)-nucleotide-binding site (NBS)-leucine rich repeat (LRR) type resistance gene by screening bacterial artificial chromosome (BAC) clones.

In this study, we report that the *de novo* genome assembly of a partial homozygous dihaploid line by TGS is a more accessible and accurate approach for gene isolation in potatoes, rather than the high-cost BAC/Yeast artificial chromosome (YAC) cloning systems. We identified the *Ry_chc_* gene as a model and revealed that it is located in the repetitive structure at the distal end of the long arm of chromosome 9.

## Materials and Methods

### Fine mapping analysis

Recombinants between two markers, RY364 and RY186 (Mori *et al.* 2011, Takeuchi *et al.* 2008), were screened from eight populations derived from crosses between *Ry_chc_*+ and *Ry_chc_*– tetraploid lines/varieties. Supplemental Table 1 lists the population name, cross combination, and the number of individuals in each population. The progeny lines used in this research were named ‘population name – individual number’.

Seeds were sown on nursery beds, and genomic DNA was extracted from leaf tips using the TPS buffer (100 mM Tris-HCl (pH 8.0), 1 M KCl, and 10 mM EDTA) (Hattori *et al.* 2007). Polymerase chain reaction (PCR) was performed according to Takeuchi *et al.* (2008). Recombinants were transplanted to 9 cm vinyl pots and cultivated until harvesting. DNA markers in the candidate region were designed based on the genomic sequence of *S. phureja* DM1-3 ver4.03 obtained from Spud DB (http://spuddb.uga.edu/index.shtml). Primer pairs were designed to amplify the intergenic or intronic region, and its band pattern was compared between ‘Konafubuki’ (*Ry_chc_*+) and ‘Hokkaikogane’ (*Ry_chc_*–) cultivars. The primers that exhibited polymorphism were used for fine mapping (Supplemental Table 2).

The total volume in the PCR assay was 10 μl, comprising 2 μl of template DNA, 5 μl of gene amplification reagent (Ampdirect Plus^®^, Shimadzu Corp., Kyoto, Japan), 0.25 U Taq DNA polymerase (BIOTAQ HS^TM^; Bioline, London, UK), and the corresponding primer pair. Thermal cycling was performed using 96- or 384-well thermal cyclers (Veriti, Applied Biosystems, Life Technologies, Carlsbad, CA, USA). The PCR conditions for RY122 (RY122-18/RY122-19), RY173 (RY173-10/RY173-27), RY186 (RY186-11/RY186-12), RY364 (Ry364-14/Ry364-19), ASRY22 (ASRY22-F/ASRY22-R), ASRY49 (ASRY49-F/ASRY49-R), ASRY63 (ASRY63-F/ASRY63-R) ASRY91 (ASRY91-F/ASRY91-R) and ASRY76 (ASRY76-F/ASRY76-R) consisted of one cycle of 10 min at 94°C; followed by 35 cycles of 30 s at 94°C, 30 s at 55°C, and 1 min at 72°C; and one final cycle of 5 min at 72°C. The PCR conditions for SZRY54 (SZRY54-5b/SZRY54-5g), SZRY183 (SZRY183-4m/SZRY183-4n), SZRY50 (SZRY50-2d/SZRY50-3f), and SZRY119 (SZRY119-F/SZRY119-R) consisted of one cycle of 10 min at 94°C; followed by 35 cycles of 30 s at 94°C, 30 s at 60°C, and 5 min at 72°C; and one final cycle of 5 min at 72°C. The PCR conditions for SZRY93 (SZRY93-4j/SZRY93-6k) consisted of one cycle of 10 min at 94°C; followed by 35 cycles of 30 s at 94°C, 30 s at 60°C, and 2 min at 72°C; and one final cycle of 5 min at 72°C. All PCR products were separated via electrophoresis on 2.0%–3.0% agarose gels in 1× TAE buffer (40 mM Tirs, 20 mM acetic acid, and 1 mM EDTA) and visualized through the SYBR Safe DNA gel stain (Invitrogen, Life Technologies, Carlsbad, CA, USA) and UV transillumination.

### Inoculation of PVY

For mechanical inoculation, a virus inoculum was prepared by grinding the infected leaves of *Nicotiana tabacum* ‘Xanthi nc’ in 10 volumes of 50 mM potassium phosphate buffer (pH 7.0) including 0.1% (w/v) sodium sulfite. The fifth and sixth leaves of potato plants at the 8–9 leaf stage (approximately 4 weeks after budding) were dusted with carborundum and mechanically inoculated using a swab. The plants were grown in a growth chamber at 22°C with 16 h of light (10,000 lux). To confirm systemic infection, the top two leaves (noninoculated upper leaves) were sampled at 21 dpi and then assayed for PVY via Triple antibody sandwich enzyme-linked immune sorbent assay (TAS-ELISA) using the ELISA Reagent Set for Potato virus Y (Agdia, Elkhar, IN, USA). Samples showing absorbance values greater than twice that of the noninoculated plants were judged to be susceptible to PVY.

### Development of a potato line for genome sequencing

We crossed the pollen of the haploid inducer *S. phureja* ‘IVP35’ to *Ry_chc_* duplex tetraploid lines ‘13082-3’ (‘Saikai 37’ × ‘Konafubuki’) and 13077-7’ (‘Saikai 35’ × ‘Hokuiku 20’). The ploidy of the progenies was analyzed using a CyFlow Ploidy Analyzer (Sysmex, Kobe, Japan), and dihaploid lines were established. Continuously, the DNA markers RY186 and RY364 were amplified via PCR. Allele dosage at the *Ry_chc_* locus was estimated from the ratio of amplification of RY364 against the internal control *aprt* in quantitative PCR from gDNA, as described by Asano and Tamiya (2013). The dihaploid ‘98H20-5’ was used as a control with heterozygous *Ry_chc_*. DNA from each progeny was extracted using DNeasy plant mini kits (QIAGEN, Venlo, Netherland). The total volume in the qPCR assay was 20 μl, comprising 2 μl of template DNA (20 ng/μl), 10 μl of SYBR Green Master Mix (Thermo Fisher Scientific, Waltham, MA, USA), and 0.1 μM forward and reverse specific primers.

### Whole-genome sequencing

For Nanopore and Illumina whole-genome sequencing, high-molecular-weight DNA was extracted from 1 g of young leaves of the line ‘184202-2’ using a Nucleobond HMW DNA kit (MACHEREY-NAGEL, Düren, Germany). We followed the manufacturer’s protocol for extraction, except for the lysing step, which was extended to 3 h. Short DNA fragments (<15 kbp) were removed from total DNA using a Short Read Eliminator (Circulomics, Pacbio, Menlo Park, CA, USA) and resuspended in TE buffer (pH 8.0). We prepared a nanopore sequencing library using SQK-LSK109 (Oxford Nanopore technology, Oxford, UK) and sequenced it three times with two FLO-MIN106D flow cells on a MinION sequencer. FAST5 data were base called using Guppy ver3.4.5 in the high accuracy mode with NVIDIA GPU (GTX1060 3GB). The adapter sequence was trimmed using the Porechop software ver0.12 (https://github.com/rrwick/Porechop). Low-quality reads (mean quality score (QS) <8) and short reads (<3000 bp) were removed using NanoFilt (De Coster *et al.* 2018). Subsequently, chimeric reads were split using yacrd (Marijon *et al.* 2020). Illumina whole-genome sequencing (150 bp paired-end) was performed on an Illumina Hiseq X Ten sequencer with Hiseq X Ten Reagent Kit v2.5 (outsourced to Eurofins Genomics). For preprocessing Illumina reads, low-quality reads (mean QS <30) and short reads (<50 bp) were filtered out using Fastp ver0.20.0 (Chen *et al.* 2018).

### *De novo* assembly

Nanopore long reads were *de novo* assembled using Flye ver2.7 (Kolmogorov *et al.* 2019) with the --nano-raw option. The obtained contigs were subjected to five runs of the Racon consensus caller ver1.4.13 (Vaser *et al.* 2017) using long reads to obtain a consensus sequence, and chimeric contigs were split using Medaka ver1.1.2 (Oxford Nanopore Technologies). Errors were further corrected three times using Pilon ver1.23 (Walker *et al.* 2014) using Illumina short read data. The assembled results were evaluated on the solanales_odb10 dataset using BUSCO ver5.3.2 (Simão *et al.* 2015). The sequences of DNA markers were searched on the contigs using NCBI-blast+ ver2.10.0 (Zhang *et al.* 2000). A dot plot analysis was conducted using D-GENIES (Cabanettes and Klopp 2018) and Gepard ver1.20 (Krumsiek *et al.* 2007).

### RNA-seq and gene prediction

Total RNA was extracted from fully expanded leaves of dihaploid ‘184202-2’ 30 days after budding using the Promega Maxwell RSC Plant RNA kit (Promega, Madison, WI, USA). Libraries were created from this RNA using the TruSeq Stranded mRNA Library Prep Kit and sequenced at 150 bp paired-end using NovaSeq6000 (outsourced to Eurofins Genomics). RNA-seq reads were extracted using Fastp for reads with QS >30 and mapped to contigs obtained via the *de novo* genome assembly of ‘184202-2’ using HISAT2.2.1 (Kim *et al.* 2019). The output of HISAT2 was converted to BAM files using Samtools ver1.9 (Li *et al.* 2009), and StringTie2 ver2.1.2 (Kovaka *et al.* 2019) was used for mRNA assembly and gene prediction. Additionally, predicted gene sequences were obtained from the GTF files using Gffread (Pertea and Pertea 2020). Transcripts found within the candidate regions were subjected to a Gene ontology analysis and BLAST search in OmicsBox (BioBam, Spain), to infer their functions.

### Cloning of candidate genes

Specific primers were manually designed in the flanking region of eight candidate genes obtained by StringTie2. The total volume of each PCR reaction was 20 μl, including 150 ng of total DNA, 10 μl of KOD One Master mix (TOYOBO, Japan), and 1 μM specific primers. PCR amplification conditions were 30 cycles of 98°C for 10 s, 65°C for 5 s, and 68°C for 1 min using an ABI9700 thermal cycler (Applied Biosystems, USA). The amplified fragments were ligated into the pCR-BluntII-TOPO vector (Thermo Fisher Scientific) and transformed into *Escherichia coli* DH5α cells. Each sublined vector was sequenced by Sanger sequencing using BigDye terminator ver3.1 (Thermo Fisher Scientific) with candidate genes specific primers shown in Supplemental Table 2.

### Data availability

The whole-genome sequencing and RNA-seq data have been deposited in the DNA Data Bank of Japan (DDBJ) repository under project number PRJDB13325. The accession numbers of each sequencing data are below. The accession numbers of nanopore whole-genome sequencing data are DRR402764, DRR402765, and DRR402766. The accession number of whole-genome sequencing data by Illumina Hiseq X pair-end is DRR402767. The accession number of mRNA sequencing data by Illumina Novaseq6000 pair-end is DRR402768. The genomic sequence of *Ry_chc_* detected in this research has been deposited as LC726345 to DDBJ.

## Results

### Fine mapping of *Ry_chc_*

**Fig. 1.**
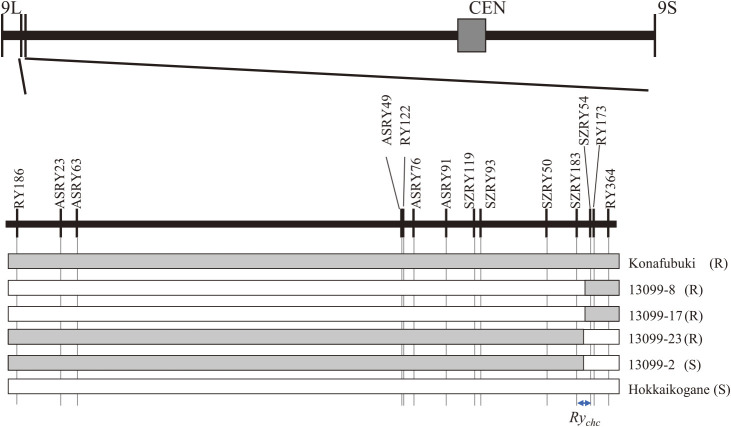
Graphical genotypes and line names of the nearest recombinant lines to *Ry_chc_*. The horizontal lines represent chromosome 9, and a physical map is shown for the *Ry_chc_* region of chromosome 9. The vertical bars represent the DNA markers. The white and gray bars indicate that the genotypes of the region are the same as those of ‘Hokkaikogane’ or ‘Konafubuki’, respectively. S or R in parentheses indicate susceptibility or resistance to PVY in each line, as determined by the inoculation test.

Before developing a *Ry_chc_* homozygous line, fine mapping was performed to narrow down the candidate region of *Ry_chc_*. In total, 62 recombinants between RY186 and RY364 were screened out from 21,364 F_1_ plants generated by eight crosses between *Ry_chc_*+ and *Ry_chc_*– lines/varieties (Supplemental Table 1). Each recombinant was genotyped using 12 newly developed DNA markers, and recombinants identified to be with recombination in the outer regions than inner DNA markers were excluded from further analysis. As a result of genotyping and phenotyping, the nearest recombinants from the population ‘13099’ obtained from the cross between ‘Sakurafubuki (*Ry_chc_*+)’ and ‘Hokkaikogane (*Ry_chc_*–)’. One recombinant (‘13099-2’) was identified between *Ry_chc_* and SZRY54; on the other side of *Ry_chc_*, three recombinants (‘13099-8’, ‘13099-17’, and ‘13099-23’) were identified between *Ry_chc_* and marker SZRY183. As a result of the fine mapping, *Ry_chc_* was delimited between SZRY183 and SZRY54 (Fig. 1, Supplemental Table 3).

### Development of a *Ry_chc_* homozygous dihaploid line

To reduce the *de novo* genome assembly difficulties caused by the heterozygous genome of autotetraploid potato, we developed a dihaploid line with homozygous *Ry_chc_* from ‘13077-7’ and ‘13082-3’, which carry duplex *Ry_chc_*. From 16 and 17 berries, respectively, we obtained 14 and 22 true seeds without embryo spots, which are indicators of triploid or tetraploid hybrids between tetraploid and ‘IVP35’ (Hutten *et al.* 1993). We generated nine and seven dihaploid individuals, respectively, nine and six of which were determined to have *Ry_chc_* from the results of the PCR amplification of RY364 and RY186 (Supplemental Table 4). Quantitative PCR revealed that only one line, named ‘184202-2’, obtained from the cross between ‘13082-3’ × ‘IVP35’, showed twice as much amplification of RY364 as the *Ry_chc_* heterozygous dihaploid line ‘98H20-5’ (data not shown). Moreover, progeny tests from a cross between the diploid cultivar ‘Inka-no-mezame’ (*Ry_chc_*–), which dose not posses RY186 and RY364, and ‘184202-2’ showed that all individuals had RY364 and RY186 (data not shown). From these results, ‘184202-2’ is confirmed as a dihaploid line with a homozygous *Ry_chc_* region.

Furthermore, ‘184202-2’ produced tubers, and had female and male fertility in the greenhouse condition (25°C–35°C) without necrosis, aerial tubers, dwarfing, or early growth arrest. It showed extreme resistance to PVY^O^ and PVY^NTN^; however, under high-temperature conditions (28°C) in the growth chamber, ‘184202-2’ formed hypersensitive reaction lesions against PVY inoculation, similar to the other varieties with *Ry_chc_* (‘Konafubuki’ and ‘Sakurafubki’) described by Ohki *et al.* (2018) (data not shown). Therefore, ‘184202-2’ had all the characteristics of *Ry_chc_* and could be used for further analysis.

### *De novo* genome assembly and gene annotation

**Fig. 2.**
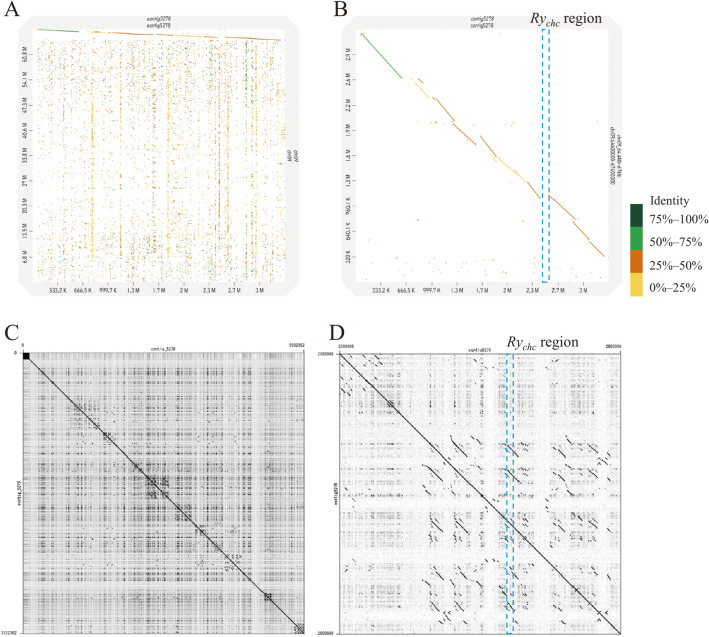
The analysis of assembled Contig5278. A: Dot plot analysis of Contig5278 against chromosome 9 of the reference genome of *S. phureja* DM1-3 v6.1, as drawn by D-GENIES. Contig5278 matched the distal end of chromosome 9 (64–67 Mb). Dot colors indicate sequence identity, as follows. Dark green: 75%–100% identity; light green: 50%–75% identity; brown: 25%–50% identity; yellow: 0%–25%. B: Enlarged view of the dot plot of Contig5278 vs. the distal end of chromosome 9 (64.4–67.6 Mb) of DM1-3 ver6.1. The light-blue box shows the *Ry_chc_* region on Contig5278. The *Ry_chc_* region is absent in the DM1-3 reference genome. C: Self-dot plot of Contig5278 drawn by Gepard-2.1 (Word length: 10, Lower color limit: Minimum, Upper color limit: Maximum, Greyscale start: Minimum). The off-diagonal dots in the plot indicate that each is a repeating sequence in Contig5278. D: Enlarged view of the self-dot plot (2.3–2.6Mb) around the *Ry_chc_* region (light-blue box area). *Ry_chc_* candidates are surrounded by highly repeated sequences.

**Fig. 3.**
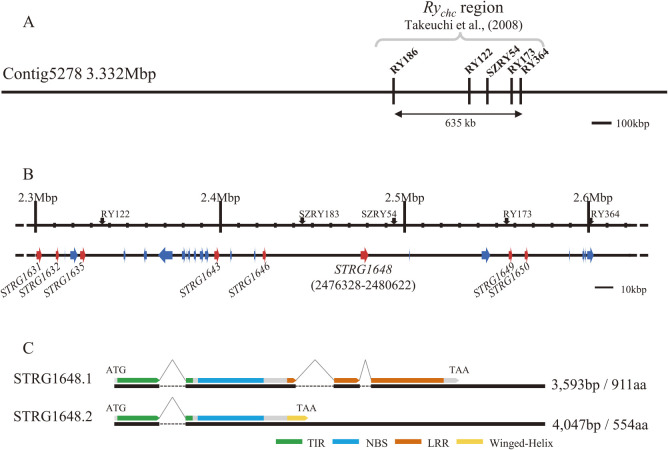
The position of *Ry_chc_* linked makers and candidate genes found on Contig5278. A: DNA markers linked to *Ry_chc_* located on the 3.3-Mb-long Contig5278. B: The positions and direction of genes predicted on Contig5278 by RNA-seq analysis from matured leaves. The red arrows indicate NBS-LRR-type resistance genes, whereas the blue arrows indicate other genes. The *Ry_chc_* candidate gene STRG1648 was predicted between SZRY183 and SZRY54. C: The structures of two splicing variants of STRG1648 and their encoded proteins. The black boxes indicate exons and the dotted lines indicate introns. The green boxes indicate the TIR domain. The light-blue boxes indicate the NBS domain. The brown boxes indicate the LRR domain. The yellow box indicates the Winged-Helix domain. STRG1648.1 encodes a 911-amino acid complete TIR-NBS-LRR-type protein with three introns. STRG1648.2 skips the second and third introns and has an early stop codon in the second exon.

Table 1 shows the statistics of NGS and TGS data used in this study. *De novo* assembly by Flye from about 12 Gb of nanopore long reads generated 9,622 contigs, with the largest contig being 9.67 Mb long and contig N50 being 352 kb (Table 2). After error polishing using Pilon v1.23 with about 80× of Illumina short reads, the assembled contigs were subjected to a BUSCO ver. 5.3.2 analysis, to assess the completeness of the assembly. In the 5,950-gene set of the solanales_odb10 dataset, 96.9% of genes were identified (83.0% were single and 13.9% were duplicated), 0.9% were fragmented, and 2.2% were missing (Table 2). Compared with the reference genomes of potatoes, the assembly of ‘184202-2’ was considered suitable for searching the *Ry_chc_* region. Also, Contig5278 was homologous to the distal end of the long arm of chromosome 9 of *S. phureja* DM1-3 v6.1 (Pham *et al.* 2020) (Fig. 2A). However, the sequence identity was under 50% around the *Ry_chc_* region in Contig5278 (Fig. 2B). A self-dot plot analysis revealed a highly repeated structure for Contig5278 (Fig. 2C), and the *Ry_chc_* region was located in a duplicated island (Fig. 2D). Next, we searched the primer sites of DNA markers linked to *Ry_chc_* in the contigs through BLAST search. Among these contigs, only Contig5278 (3.3 Mb) included markers RY186 and RY364 linked to *Ry_chc_*; RY186 and RY364 were contained in a region of approximately 635 kb (Fig. 3A). Moreover, sites of other DNA markers designed in the fine mapping were found between RY186 and RY364.

### Gene annotation and candidate genes of *Ry_chc_*

*Ry_chc_* induces resistance against PVY inoculation on leaves, which should be detected via gene expression analysis in leaves. RNA-seq analysis from matured leaves of ‘184202-2’ predicted 27 genes between the 2.3–2.6 Mb point and the *Ry_chc_* candidate region (Fig. 3B). “STRG” is the default prefix of StringTie2. A BLAST analysis led to the annotation of eight of these 27 genes as *Rpi-vnt1*-like (CC-NBS-LRR) or tobacco mosaic virus (TMV) resistance gene *N*-like (TIR-NBS-LRR)-type resistance genes (Table 3). However, six of the eight resistance candidate genes had early stop codons in the sequence and encoded truncated proteins; therefore, these genes were not likely to be the *Ry_chc_* gene. The genome sequences of each candidate genes were shown in Supplemental Table 5. Furthermore, based on the locus position of *Ry_chc_*, as inferred based on fine mapping and the structures of these candidate genes, STRG1648 was expected to be *Ry_chc_*.

**Fig. 4.**
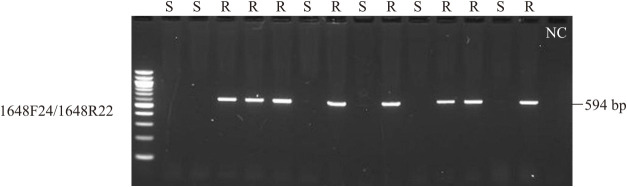
PCR amplification using the 1648F24/1648R22 primer set in recombinant lines. The phenotypes of the recombinants are shown above the lanes (R: resistant, S: susceptible). Lanes 1–15: NEB 100 bp DNA Ladder marker; 11023-27(S); 13099-2(S); 13099-5(R); 13099-8(R); 13099-17(R); 13099-22(S); 13099-23(R); 13099-31(S);13096-5(R); 14116-1(S); 14116-5(R); 14116-11(R); Hokkaikogane(S); Sakurafubuki(R); negative control. The 594-bp band is perfectly correlated to the PVY resistance phenotype.

An RNA-seq analysis showed that STRG1648 had two splicing variants (Fig. 3C). The STRG1648.1 variant was predicted to have three introns in the transcript, encoding a 911-amino acid TIR-NBS-LRR-type protein (Table 3). In turn, the STRG1648.2 variant was predicted to skip the second and third introns and had a stop codon at the start of the LRR region; thus, the STRG1648.2 variant encoded a truncated Winged-Helix domain, instead of the LRR domain (Fig. 3C). Additionally, the presence/absence of STRG1648 amplification via PCR (1648F24/1648R22) correlated perfectly with the PVY-resistant phenotype (Fig. 4, Supplemental Fig. 3, Supplemental Table 3).

### Nucleotide accuracy of the *de novo* assembly

The sequence of STRG1648 obtained from the *de novo* assembly perfectly matched the sequence of the PCR amplicon obtained using the primer pair STRG1648f-F/STRG1648f-R, as determined via Sanger sequencing (Supplemental Fig. 1). This result indicates that the nucleotide determination accuracy after polishing by Illumina reads was very high. Moreover, Supplemental Fig. 1 shows the comparison of the genome sequence of the STRG1648 gene with the *Ry_chc_* sequence reported by Li *et al.* (2022). The nucleotide identity between *Ry_chc_* and STRG1648 was 98% (4108/4176 bp) for the entire gene; therefore, we concluded that STRG1648 was the *Ry_chc_* gene. Additionally, single nucleotide polymorphisms, insertions, and deletions were detected between two *Ry_chc_* sequences (Supplemental Fig. 1).

## Discussion

### Long-read-based de novo assembly of the *Ry_chc_* homozygous dihaploid

The *Ry_chc_* region was estimated to be located in the NBS-LRR cluster with many repeat sequences because the reference genome DM1-3 ver4.03 (Xu *et al.* 2011), which was reconstructed by Sanger and Illumina sequencing, contains many assembly gaps in the *Ry_chc_* region. To assemble contigs that are sufficiently long to combine the results from the fine mapping analysis, we chose *de novo* assembling using nanopore sequencing technology and error polishing with Illumina high-quality short reads. Moreover, the *Ry_chc_* homozygous line ‘184202-2’ was developed using the dihaploid induction technique for genome sequencing.

As a result of the *de novo* assembly using nanopore long reads and error polishing by Illumina reads, we generated a 3.3-Mb-long contig (Contig5278) of this challenging region (Fig. 3). In Contig5278, one TIR-NBS-LRR-type resistance gene, named STRG1648, was identified between the closest markers, SZRY183 and SZRY54. Its sequence exhibited high homology with that of *Ry_chc_* reported by Li *et al.* (2022), and the presence/absence of STRG1648 was perfectly correlated with the PVY resistance phenotype; therefore, we concluded that STRG1648 was the *Ry_chc_* gene derived from *S. chacoense* ‘w84’. This strategy provided sufficient results to combine with the fine mapping results and showed that it is not necessary to construct BAC clones for map-based cloning in potatoes. Moreover, after error correction using short reads, the assembled nucleotide sequences of the *Ry_chc_* gene (STRG1648) matched perfectly with the sequences of its PCR product, as determined by Sanger sequencing. This result implies that the contigs obtained in this study were sufficiently accurate for searching not only SCAR markers but also SNP or SSR markers.

Furthermore, in recent years, long-read sequencing technologies with base-call accuracies of 99% or better have also emerged, thus solving highly heterozygous genomes, such as the RH line of diploid potato (Zhou *et al.* 2020), the commercial tetraploid cultivar ‘Otava’ (Sun *et al.* 2022), and 44 diploid potato wild species (Tang *et al.* 2022). However, such haplotype-aware assemblies still require a multi-steps assembling approach containing genetic mapping on a sequenced F_2_ population, 20x–40x of the accurate long reads of Circular Consensus Sequencing or HiFi sequencing (Pacific Biosciences) and combinations of several technologies, such as Hi-C or Bionano (Zhou *et al.* 2020). From the point of view of cost, our approach with low cost Nanopore long reads is a simpler way to obtain target sequences.

### *Ry_chc_* is a TIR-NBS-LRR-type resistance gene

*Ry_chc_* (STRG1648) was predicted to encode a protein consisting of 911 amino acid residues of the TIR-NBS-LRR type, similar to that reported by Li *et al.* (2022). Additionally, between two *Ry_chc_* sequences, some nucleotide variations were detected; therefore, these two *Ry_chc_* genes are likely in a homologous gene with similar functions that are maintained and are considered to be within the *Ry_chc_* gene family.

In this study, we detected two splicing variants of *Ry_chc_*, one of which had second and third introns (Fig. 3C). For resistance against the TMV induced by the *N* gene derived from *N. glutinosa*, splicing variants of *N* transcripts containing first and second introns are required because these splicing variants enhance the expression of the *N* gene and regulate N protein accumulation (Ikeda *et al.* 2021). Two splicing variants predicted for *Ry_chc_* may be required for the stable and robust resistance afforded by *Ry_chc_* by playing a role in controlling *Ry_chc_* expression and protein accumulation levels during PVY infection as the *N* gene.

PVY accumulates mutations and recombinations in its genome continuously (Ogawa *et al.* 2008, 2012, Rodriguez-Rodriguez *et al.* 2020); therefore, the possibility of PVY isolates overcoming *Ry_chc_* resistance cannot be ruled out. To evaluate this possibility, further studies are warranted to clarify in detail which proteins of PVY are recognized by *Ry_chc_* as Avrs and what resistance mechanisms are at work.

### The origin of *Ry_chc_*

The number of NBS-LRR-type disease resistance genes has generally increased because of events such as large-scale tandem repeats caused by unequal crossing over. The newly duplicated genes are relieved of selection pressure and accumulate mutations, resulting in the recognition of various pathogens(Han and Tsuda 2022). The highly repeated sequence located around *Ry_chc_* in Contig5278 suggests that many tandem duplication events occurred in the distal end of the long arm of chromosome 9 (Fig. 2). Moreover, a sequence comparison between Contig5278 and the reference genomes of *S. chacoense* ‘M6’ (Leisner *et al.* 2018) and *S. tuberosum* ‘Solyntus’ (Van Lieshout *et al.* 2020) showed the presence of various structural variants, such as inversions, duplications, and deletions (Supplemental Fig. 2).

Conversely, the accumulation of random mutations on duplicated resistance genes may induce an autoimmune response by recognizing the proteins they produce, which in turn trigger not only programmed cell death but also a systemic hypersensitive reaction. In such cases, mutations in the promoter region or some early stop codons appear to inactivate harmful resistance genes (Barragan *et al.* 2021). The fact that six of the eight disease resistance genes annotated in the *Ry_chc_* locus have early stop codons suggests the existence of such natural selection.

Li *et al.* (2022) suggested that *Ry_chc_* may have rapidly accumulated mutaions and obtained the ability to recognize PVY as the PVY geneome diversified and subsequently spread and maintained in the *S. chacoense* population. Traces of duplication events revealed in our study suggest that the region of *Ry_chc_* has experienced multiple duplication events and may support the view of that the mutation expanded under the selection pressure of PVY described in Li *et al.* (2022). However, it is not possible to determine from these results when *Ry_chc_* was emerged in the duplication events and when it acquired the ability of PVY recognition.

### Conclusion

In this study, the PVY resistance gene *Ry_chc_* was detected by map-based cloning using traditional haploid induction techniques and *de novo* genome assembly with long reads. Compared with the previously reported *Ry_chc_* gene, the sequence of the *Ry_chc_* identified in this study included several mutations, indicating that the *Ry_chc_* family exhibits some diversity. The repeated sequences detected on Contig5278 were thought of as traces of the gene duplication events that led to the *Ry_chc_* gene. Furthermore, the sequence of contigs obtained in this study was accurate, implying that any type of DNA marker can be detected. Also, further research on how *Ry_chc_* recognizes PVY and initiates the resistance cascade will determine the future of *Ry_chc_* use in PVY resistance potato breeding. Additionally, our gene isolation approach will also work well in the search for other valuable genes in potatoes.

## Author Contribution Statement

K. Akai carried out the development of the dihaploid clone, whole-genome sequence, *de novo* assembly, and gene annotation. K. Asano, CS, TS, and TT designed primers for the fine mapping and screened recombinant lines. ES and ST carried out the development of *Ry_chc_* duplex tetraploid lines. TO carried out PVY inoculation test and cloning of candidate genes. All authors were involved in improving this manuscript.

## Supplementary Material

Supplemental Figures

Supplemental Tables

## Figures and Tables

**Table 1.** Statistics of the genome sequencing data used for *de novo* assembly and RNA-seq data

Nanopore WGS	Total bases (bp)	12,900,979,133
	Total reads	725,512
	Average read length (bp)	17,781.9
	Longest read length (bp)	250,644
	Mean QS	11.5
	Read N50 (bp)	34,181
Illumina WGS	Total bases (bp)	82,236,131,442
	Total reads	544,610,142
	Read length (bp)	150
	QS >30	93.6%
	QS >20	97.2%
Illumina RNA-seq	Total bases (bp)	8,412,300,300
	Total reads	56,082,002
	Read length (bp)	150
	QS >30	94.3%
	QS >20	97.6%

**Table 2.** Statistics of the genome assembly of ‘184202-2’

	Genome assembly			
	*S. tuberosum* 184202-2	*S. phureja* DM1-3 v4.04	*S. phureja* DM1-3 v6.01	*S. chacoense* M6 v4.1
Parameters	(this study)	(Hardigan *et al.* 2016)	(Pham *et al.* 2020)	(Leisner *et al.* 2018)
Methods	Nanopore long reads and Illumina	Illumina and Sanger	Nanopore long reads	Illumina
Total assembly size (Mb)	1,091.1	884.1	741.6	825.7
Number of contigs	9,622	170,833	1,382	–
Number of scaffolds	–	14,853	288	8,260
Largest contig length (bp)	9,678,383	–	–	–
Average contig length (bp)	113,393	–	–	–
Largest scaffold length (bp)	–	111,078,864	88,591,686	7,385,816
Average scaffold length (bp)	–	63,150,592	2,574,948	99,972
Contig N50 (bp)	352,213	170,833	17,312,182	–
Scaffold N50 (bp)	–	1,344,915	59,670,755	713,601
BUSCO ver5.3.2 with solanales_odb10 dataset				
Total	5,950	5,950	5,950	5,950
Complete	96.9%	98.6%	98.7%	89.8%
single	83.0%	96.3%	96.4%	87.5%
duplicated	13.9%	2.3%	2.3%	2.3%
Fragmented	0.9%	0.2%	0.1%	0.2%
Missing	2.2%	1.2%	1.2%	10.0%

**Table 3.** Predicted genes in the *Ry_chc_* region and their properties

Gene Name	BLAST Description	Start position on Contig5278 (bp)	Gene length (nt)	Transcript length (nt)	Protein length (aa) from Kozak sequence	Gene type
STRG1629	60S acidic ribosomal protein P0	2,292,859	1,006	1,006	280	
STRG1627	---NA---	2,298,771	911	911	1	
STRG1631	Rpi-vnt1-like protein	2,300,618	2,895	2,895	864	CC-NBS-LRR
STRG1630	Late blight resistance protein, putative	2,307,144	297	297	2	
STRG1632	Rpi-vnt1-like protein	2,310,970	1,783	1,783	20	
STRG1633	Hypothetical protein SDM1_42t00007	2,316,039	210	210	No ATG found	
STRG1634	---NA---	2,321,599	1,326	557	3	
STRG1635	Rpi-vnt1-like protein	2,324,216	3,016	2,917	664	NBS-LRR
STRG1636	Protein ECERIFERUM 26-like	2,348,256	1,080	1,080	21	
STRG1637	Chaperone protein dnaj 20, chloroplastic-like	2,358,580	1,840	737	2	
STRG1642	Serine/arginine repetitive matrix protein 2	2,365,985	8,369	4,138	900	
				4,435	900	
				4,054	900	
				3,669	900	
				3,694	900	
				3,665	900	
STRG1638	Small subunit processome component 20 homolog	2,382,753	1,154	734	2	
STRG1639	Small subunit processome component 20 homolog	2,389,043	2,096	1,435	405	
STRG1640	Small subunit processome component 20 homolog	2,391,240	1,084	996	330	
STRG1641	Small subunit processome component 20 homolog	2,393,727	369	369	61	
				1,189	258	
STRG1643	TMV resistance protein N-like	2,397,081	2,677	2,677	49	LRR
STRG1644	Deacetoxyvindoline 4-hydroxylase-like	2,407,359	942	456	143	
STRG1645	Small subunit processome component 20 homolog	2,418,710	816	816	10	
STRG1646	TMV resistance protein N-like	2,423,296	2,043	2,043	49	LRR
STRG1648	TMV resistance protein N-like	2,476,328	4,294	3,593	911	TIR-NBS-LRR
				4,047	554	TIR-NBS-WingedHelix
STRG1647	---NA---	2,502,697	348	348	22	
STRG1651	Small subunit processome component 20 homolog	2,541,640	4,981	3,070	200	
				2,523	200	
STRG1649	TMV resistance protein N-like	2,556,562	2,067	1,820	552	TIR-NBS-WingedHelix
STRG1650	TMV resistance protein N-like	2,565,200	2,003	1,914	373	partial LRR
STRG1652	Protein RALF-like 32	2,589,214	467	467	8	
STRG1653	Probable magnesium transporter NIPA2	2,597,949	603	360	80	
STRG1654	Stress response protein NST1-like	2,600,362	2,366	2,366	48	
