# Supplementary material for: De novo genome assembly of the partial homozygous dihaploid potato identified PVY resistance gene (Rychc) derived from Solanum chacoense
Source: Breed Sci. 2023 Apr 13;73(2):168–79. doi: 10.1270/jsbbs.22078 (PMC10316315; doi:10.1270/jsbbs.22078)
Supplement: Supplementary file 1 — Supplemental Figures [file 73_168_s1.pdf]

doi: 10.1270/jsbbs.22078

```
STRG1648_denovo        CTTGTGGATATATAAAGAGCTGCTTCACATCTCTATTTAATTAATTAAACAAAAAAATTG
STRG1648_sangar        CTTGTGGATATATAAAGAGCTGCTTCACATCTCTATTTAATTAATTAAACAAAAAAATTG
Rychc_Li_etal_2022     ------------------------------------------------------------

STRG1648_denovo        ATTAAAAATCCCTTTAATATATTGTCATCAAATATGAATACTCAAGGAGAATCATCTTCT
STRG1648_sangar        ATTAAAAATCCCTTTAATATATTGTCATCAAATATGAATACTCAAGGAGAATCATCTTCT
Rychc_Li_etal_2022     ---------------------------------ATGAATACTCAAGGAGAATCATCTTCT
                                                        ***************************

STRG1648_denovo        TCTTCCAACTTATGTTATGATGTGTTTCTCAGTTTCAGAGGTGAAGATACTCGCAAAAAC
STRG1648_sangar        TCTTCCAACTTATGTTATGATGTGTTTCTCAGTTTCAGAGGTGAAGATACTCGCAAAAAC
Rychc_Li_etal_2022     TCTTCCAACTTATGTTATGATGTGTTTCTCAGTTTCAGAGGTGAAGATACTCGCAAAAAC
                       ************************************************************

STRG1648_denovo        TTCATTGATCATCTTTATTTCCGATTATGTCAAGTCGGAGTTAATACTTTCATAGATGAT
STRG1648_sangar        TTCATTGATCATCTTTATTTCCGATTATGTCAAGTCGGAGTTAATACTTTCATAGATGAT
Rychc_Li_etal_2022     TTCATTGATCATCTTTATTTCCGATTATGTCAAGTCGGAGTTAATACTTTCATAGATGAT
                       ************************************************************

STRG1648_denovo        GAGGAATTGAGAAAGGGAGACGTCATTTCAAACAAACTTGACAAAGCAATTGAACAATCT
STRG1648_sangar        GAGGAATTGAGAAAGGGAGACGTCATTTCAAACAAACTTGACAAAGCAATTGAACAATCT
Rychc_Li_etal_2022     GAGGAATTGAGAAAGGGAGACGTCATATCAAAGAAACTTGACAAAGCAATTGAACAATCT
                       ************************** ***** ***************************

STRG1648_denovo        AGAATTGCCATTGTTGTTTTCTCGAAAAATTATGCTTCGTCTAGTTGGTGTCTTGATGAA
STRG1648_sangar        AGAATTGCCATTGTTGTTTTCTCGAAAAATTATGCTTCGTCTAGTTGGTGTCTTGATGAA
Rychc_Li_etal_2022     AGAATTGCCATTGTTGTTTTCTCGAAAAATTATGCTTCGTCTAGTTGGTGTCTTGATGAA
                       ************************************************************

STRG1648_denovo        CTTGTCAAAATTCTCGATTGCAAAGAGAGGTTAAATCAGGTAGTTTTGCCTATTTTCTAT
STRG1648_sangar        CTTGTCAAAATTCTCGATTGCAAAGAGAGGTTAAATCAGGTAGTTTTGCCTATTTTCTAT
Rychc_Li_etal_2022     CTTGTCAAAATTCTCGATTGCAAAGAGAGGTTAAATCAGGTAGTTTTGCCTATTTTCTAT
                       ************************************************************

STRG1648_denovo        GATGTTGATCCTTCTCAAGTGCGAAGGCAAACTGGATCCTTTGACGAAGCTTTGGAAAAA
STRG1648_sangar        GATGTTGATCCTTCTCAAGTGCGAAGGCAAACTGGATCCTTTGACGAAGCTTTGGAAAAA
Rychc_Li_etal_2022     GATGTTGATCCTTCTCAAGTGCGAAGGCAAACTGGATCCTTTGGCGAAGCTTTGTCAAAA
                       ******************************************* **********  ****

STRG1648_denovo        CACCAGGAACGATTGATTGGAGCTGAAAGAATGGAAAAGTGGAAAGCTGCACTTACTAAA
STRG1648_sangar        CACCAGGAACGATTGATTGGAGCTGAAAGAATGGAAAAGTGGAAAGCTGCACTTACTAAA
Rychc_Li_etal_2022     CACAAGGGACGATTAGTTGGAGCTGAAAGAATGGAAAAGTGGAAAGCTGCACTTACTGAA
                       *** *** ******  ***************************************** **

STRG1648_denovo        GCAGCAAATTTATCTGGATGGGATTTGAGAAATGTTGCTGATGGG-TAAGACTTCTTAAT
STRG1648_sangar        GCAGCAAATTTATCTGGATGGGATTTGAGAAATGTTGCTGATGGG-TAAGACTTCTTAAT
Rychc_Li_etal_2022     GCAGCAAATTTATCTGGATGGGATTTFAFAAATATTGCTGATGGGGTAAGACTTCTTAAT
                       ************************** * **** *********** **************

STRG1648_denovo        TCAACTATTAAATTTTGAATTTAAGCATTTGCCTAAGCTTATAAGTTGGTTAATTTTGGA
STRG1648_sangar        TCAACTATTAAATTTTGAATTTAAGCATTTGCCTAAGCTTATAAGTTGGTTAATTTTGGA
Rychc_Li_etal_2022     TCAACTATTAAATTTTGAATTTAAGCGCTTGCCTAAGCTTATAAGTTGCTTAATTATGGA
                       **************************  ******************** ****** ****

STRG1648_denovo        ACATTGTAAAGTCTTTTAATATTTCTTAAATTTCGTATCAAATCAAACTGTGTCGATTGG
STRG1648_sangar        ACATTGTAAAGTCTTTTAATATTTCTTAAATTTCGTATCAAATCAAACTGTGTCGATTGG
Rychc_Li_etal_2022     ACATTGTAAAGTTTTTTAATATTT-TTAAATTTCGTATCAAATCAAACTGTGTCGATTGG
                       ************ *********** ***********************************

STRG1648_denovo        GACAAGGAATAATCTCCATTCTATAAACTCCGTTATCTTTTGGCTATTGGCTTTTATTTT
STRG1648_sangar        GACAAGGAATAATCTCCATTCTATAAACTCCGTTATCTTTTGGCTATTGGCTTTTATTTT
Rychc_Li_etal_2022     GACAAGGAATAATCTCCATTCTATAAACTCTGTTATCTTTTGGCTATTGGCTTTTATTTT
                       ****************************** *****************************

STRG1648_denovo        GTTCATCAAATATTTTAAAATACTTAAGTATTTATTGTTGCAAAAACTGTAGGCATGAAT
STRG1648_sangar        GTTCATCAAATATTTTAAAATACTTAAGTATTTATTGTTGCAAAAACTGTAGGCATGAAT
Rychc_Li_etal_2022     GTTCATCAAATATTTTAAAATACTTAAGTATTTATTGTTGCAAAAACTGTAGGCATGAAT
                       ************************************************************
```

Supplemental Fig. 1 (Continued)

```
STRG1648_denovo       CAAGGTTTATTGAAAGTATTATAAAACAAGTTCTGCAAGAGGTTAACCAGACACCTCTAG
STRG1648_sangar       CAAGGTTTATTGAAAGTATTATAAAACAAGTTCTGCAAGAGGTTAACCAGACACCTCTAG
Rychc_Li_etal_2022    CAAAGTTTATTGAGAGTGTTATAAAACAAGTTCTGCAAGAGGTTAACCAGACACCTCTAG
                      *** ********* *** ******************************************

STRG1648_denovo       ATGTTGCTCATTACCCAATTGGATTAGATTCTCCTATCAAACATATAGAGGTGTTACTGC
STRG1648_sangar       ATGTTGCTCATTACCCAATTGGATTAGATTCTCCTATCAAACATATAGAGGTGTTACTGC
Rychc_Li_etal_2022    ATGTTGCTCATTACCCAATTGGATTAGATTCTCCTATCAAACATATAGAGGTGTTACTGC
                      ************************************************************

STRG1648_denovo       AAAGTGGATGTGAGCATGAAGTTCGCATGGTTGGTATATGTGGCATTGGTGGAATTGGAA
STRG1648_sangar       AAAGTGGATGTGAGCATGAAGTTCGCATGGTTGGTATATGTGGCATTGGTGGAATTGGAA
Rychc_Li_etal_2022    AAAGTGGATATGAGCATGAACTTCGCATGATTGGTATATGTGGCATTGGTGGAATTGGAA
                      ********* ********** ******** ******************************

STRG1648_denovo       AAACAACTTTGGCAAAAGAAATCTATAATCGAATATTTCAACAGTTTGATGGTAGTTGCT
STRG1648_sangar       AAACAACTTTGGCAAAAGAAATCTATAATCGAATATTTCAACAGTTTGATGGTAGTTGCT
Rychc_Li_etal_2022    AAACAACTTTCGCAAAAGCTATCTATAATCGAATATTTCAACAGTTTGATGGTAGTTGCT
                      ********** *******  ****************************************

STRG1648_denovo       TCCTTTCTGACATTAGATCAAAAACTGAAGAATCGGGTCTAATCAAGCTTCAAGAGAAAC
STRG1648_sangar       TCCTTTCTGACATTAGATCAAAAACTGAAGAATCGGGTCTAATCAAGCTTCAAGAGAAAC
Rychc_Li_etal_2022    TCCTTTCTGACATTAGATCAAAAACTGAAGAATCGGGTCTAATCAAGCTTCAAGAGAAAC
                      ************************************************************

STRG1648_denovo       TACTTTATCAAATCCTCAAAACTAAGGAATTTGAAGTTGATAGTGTTGCTGAAGGTGTTA
STRG1648_sangar       TACTTTATCAAATCCTCAAAACTAAGGAATTTGAAGTTGATAGTGTTGCTGAAGGTGTTA
Rychc_Li_etal_2022    TACTTTATCAAATCCTCAAAACTAAGGAATTTGAAGTTGATAGTGTTGCTGAAGGTGTTA
                      ************************************************************

STRG1648_denovo       ATCTCATCAAAGCAAGACTTGGGTCTCAGAAGGTTCTAATTGTTCTTGATGACGTGGATC
STRG1648_sangar       ATCTCATCAAAGCAAGACTTGGGTCTCAGAAGGTTCTAATTGTTCTTGATGACGTGGATC
Rychc_Li_etal_2022    ATCTCATCAAAGCAAGACTTGGGTCTCAGAAGGTTCTAATTGTTCTTGATGACGTGGATC
                      ************************************************************

STRG1648_denovo       ATAGAAGCCAATTAGAATCCTTAACAAGAGAAAGAAGTTGGTTTGGCTTAGGTAGTGTAA
STRG1648_sangar       ATAGAAGCCAATTAGAATCCTTAACAAGAGAAAGAAGTTGGTTTGGCTTAGGTAGTGTAA
Rychc_Li_etal_2022    ATAGAAGCCAATTAGAATCCTTAACAAGAGAAAGAAGTTGGTTTGGCTTAGGTAGTGTAA
                      ************************************************************

STRG1648_denovo       TAATTATTACAACCCGAGATGAACATTTGCTATATGGGCTTACAACAAGTGAGATATACC
STRG1648_sangar       TAATTATTACAACCCGAGATGAACATTTGCTATATGGGCTTACAACAAGTGAGATATACC
Rychc_Li_etal_2022    TAATTATTACAACCCGAGATGAACATTTGCTATATGGGCTTACAACAAGTGAGATATACC
                      ************************************************************

STRG1648_denovo       AGGCCAAACTTTTAAATGACAAGGAAGCCCAACAACTTTTTTCTTGTCATGCTTTTAACT
STRG1648_sangar       AGGCCAAACTTTTAAATGACAAGGAAGCCCAACAACTTTTTTCTTGTCATGCTTTTAACT
Rychc_Li_etal_2022    AGGCCAAACTTTTAAATGACAAGGAAGCCCAACAACTTTTTTCTTGTCATGCTTTTAACT
                      ************************************************************

STRG1648_denovo       GTTTTTCTCCACCACAAGAATATGTTGAACTGGCACAAGACATAATAAAATATTCAGGTG
STRG1648_sangar       GTTTTTCTCCACCACAAGAATATGTTGAACTGGCACAAGACATAATAAAATATTCAGGTG
Rychc_Li_etal_2022    GTTTTTCTCCACCACAAGAATATCTTAAACTGGCACAAGACATAATAAAATATTCAGGTG
                      *********************** ** *********************************

STRG1648_denovo       GGCTACCATTAGCTCTTGTGACATTGGGGTCACATCTGCAAGGGAGATCCGTTGAAGAAT
STRG1648_sangar       GGCTACCATTAGCTCTTGTGACATTGGGGTCACATCTGCAAGGGAGATCCGTTGAAGAAT
Rychc_Li_etal_2022    GGCTACCATTAGCTCTTGTGACATTGGGGTCACATTTGCAAGGGAGATCCGTTGAAGAAT
                      *********************************** ************************

STRG1648_denovo       GGAGATACGAGTTCAAAAAACTAAAAGCAATTCCTCATGGTGATATTCAAAAGATTCTCA
STRG1648_sangar       GGAGATACGAGTTCAAAAAACTAAAAGCAATTCCTCATGGTGATATTCAAAAGATTCTCA
Rychc_Li_etal_2022    GGAGATACGAGTTCAAAAAACTAAAAGCAATTCCTCATGGTGATATTCAAAAGATTCTCA
                      ************************************************************

STRG1648_denovo       AGATAAGCTTTGATGGACTTGACGCCAATACTCAGAGTGTTTTCCTTGATATCGCATTTG
STRG1648_sangar       AGATAAGCTTTGATGGACTTGACGCCAATACTCAGAGTGTTTTCCTTGATATCGCATTTG
Rychc_Li_etal_2022    AGATAAGTTTTGATGGACTTGACGCGAATACTCAGAGTGTTTTCCTTGATATCGCATTTG
                      ******* ***************** **********************************
```

Supplemental Fig. 1 (Continued)

```
STRG1648_denovo      TAATTATTACAACCCGAGATGAACATTTGCTATATGGGCTTACAACAAGTGAGATATACC
STRG1648_sangar      TAATTATTACAACCCGAGATGAACATTTGCTATATGGGCTTACAACAAGTGAGATATACC
Rychc_Li_etal_2022   TAATTATTACAACCCGAGATGAACATTTGCTATATGGGCTTACAACAAGTGAGATATACC
                     ************************************************************

STRG1648_denovo      AGGCCAAACTTTTAAATGACAAGGAAGCCCAACAACTTTTTTCTTGTCATGCTTTTAACT
STRG1648_sangar      AGGCCAAACTTTTAAATGACAAGGAAGCCCAACAACTTTTTTCTTGTCATGCTTTTAACT
Rychc_Li_etal_2022   AGGCCAAACTTTTAAATGACAAGGAAGCCCAACAACTTTTTTCTTGTCATGCTTTTAACT
                     ************************************************************

STRG1648_denovo      GTTTTTCTCCACCACAAGAATATGTTGAACTGGCACAAGACATAATAAAATATTCAGGTG
STRG1648_sangar      GTTTTTCTCCACCACAAGAATATGTTGAACTGGCACAAGACATAATAAAATATTCAGGTG
Rychc_Li_etal_2022   GTTTTTCTCCACCACAAGAATATCTTAAACTGGCACAAGACATAATAAAATATTCAGGTG
                     *********************** ** *********************************

STRG1648_denovo      GGCTACCATTAGCTCTTGTGACATTGGGGTCACATCTGCAAGGGAGATCCGTTGAAGAAT
STRG1648_sangar      GGCTACCATTAGCTCTTGTGACATTGGGGTCACATCTGCAAGGGAGATCCGTTGAAGAAT
Rychc_Li_etal_2022   GGCTACCATTAGCTCTTGTGACATTGGGGTCACATTTGCAAGGGAGATCCGTTGAAGAAT
                     *********************************** ************************

STRG1648_denovo      GGAGATACGAGTTCAAAAAACTAAAAGCAATTCCTCATGGTGATATTCAAAAGATTCTCA
STRG1648_sangar      GGAGATACGAGTTCAAAAAACTAAAAGCAATTCCTCATGGTGATATTCAAAAGATTCTCA
Rychc_Li_etal_2022   GGAGATACGAGTTCAAAAAACTAAAAGCAATTCCTCATGGTGATATTCAAAAGATTCTCA
                     ************************************************************

STRG1648_denovo      AGATAAGCTTTGATGGACTTGACGCCAATACTCAGAGTGTTTTCCTTGATATCGCATTTG
STRG1648_sangar      AGATAAGCTTTGATGGACTTGACGCCAATACTCAGAGTGTTTTCCTTGATATCGCATTTG
Rychc_Li_etal_2022   AGATAAGTTTTGATGGACTTGACGCGAATACTCAGAGTGTTTTCCTTGATATCGCATTTG
                     ******* ***************** **********************************

STRG1648_denovo      CCTTCCATGGTTGTGATGAGGATGAAGTTACCAAAACATTAAATGCGTGTGGTTTTTATT
STRG1648_sangar      CCTTCCATGGTTGTGATGAGGATGAAGTTACCAAAACATTAAATGCGTGTGGTTTTTATT
Rychc_Li_etal_2022   CCTTCCATGGTTGTGATGAGGATGAAGTTACCAAAACATTAAATCCGTGTGGTTTTTATT
                     ******************************************** ***************

STRG1648_denovo      CTGAAAGTGCAATTTCAACCTTAGTACAAAGGAACTTGGTCCAAAGGAATAGGCCTCGTT
STRG1648_sangar      CTGAAAGTGCAATTTCAACCTTAGTACAAAGGAACTTGGTCCAAAGGAATAGGCCTCGTT
Rychc_Li_etal_2022   CTGAAAGTGCAATTTCAACCTTAGTACAAAGGAACTTGGTCCAAAGGAATAGGCCTCGTT
                     ************************************************************

STRG1648_denovo      TGGTGATGCATGATCTAGTGCAGGAAATGGGAAGAGAAATCGTTCGCATGGAATCTCAAG
STRG1648_sangar      TGGTGATGCATGATCTAGTGCAGGAAATGGGAAGAGAAATCGTTCGCATGGAATCTCAAG
Rychc_Li_etal_2022   TGGTGATGCATGATCTAGTGCAGGAAATGGGAAGAGAAATCGTTCGCATGGAATCTCAAG
                     ************************************************************

STRG1648_denovo      ACCCTGGAAAACGGAGTAGATTGTTCAACCCTCAAGAAGTCATTGATGTTCTACAAGGAA
STRG1648_sangar      ACCCTGGAAAACGGAGTAGATTGTTCAACCCTCAAGAAGTCATTGATGTTCTACAAGGAA
Rychc_Li_etal_2022   ACCCTGGAAAACGGAGTAGATTGTTCAACCCTCAAGAAGTCATTGATGTTCTACAAGGAA
                     ************************************************************

STRG1648_denovo      ATAAAGTCAGTAAATTTCATTATCTTTATCTTGGTTATACTTCTTCTTCCTTTTTTTCAT
STRG1648_sangar      ATAAAGTCAGTAAATTTCATTATCTTTATCTTGGTTATACTTCTTCTTCCTTTTTTTCAT
Rychc_Li_etal_2022   ATAAAGTCAGTAAATTTCATTATCTTTATCTTGGTTATACTTCTTCTTCCTTTTTTTCAT
                     ************************************************************

STRG1648_denovo      TTATATTGATTTACATTTGCTTATAATTTATGCATTTGTGCTTAAATCTTCTCCGGCTAG
STRG1648_sangar      TTATATTGATTTACATTTGCTTATAATTTATGCATTTGTGCTTAAATCTTCTCCGGCTAG
Rychc_Li_etal_2022   TTATATTGATTTACATTTGCTTATAATTTATGCATTTGTGCTTAAATCTTCTCCGGCTAG
                     ************************************************************

STRG1648_denovo      TAAGTATCTCTAGAAGTTTGAACCATTCTGTACTTGAAATGAACATTTACTGAAGTCTAA
STRG1648_sangar      TAAGTATCTCTAGAAGTTTGAACCATTCTGTACTTGAAATGAACATTTACTGAAGTCTAA
Rychc_Li_etal_2022   TAAGTATCTCTAGAAGTTTGAACCATTCTGTACTTGAAATGAACATTTACTGAAGTCTAA
                     ************************************************************

STRG1648_denovo      ACACTCAAACTATCAAAGACAAAATTATCAAATAAATATCCTTTAGTTGGATATAATGTG
STRG1648_sangar      ACACTCAAACTATCAAAGACAAAATTATCAAATAAATATCCTTTAGTTGGATATAATGTG
Rychc_Li_etal_2022   ACACTCAAACTATCAAAGACAAAATTATCAAATAAATATCCTTTAGTTGGATATAATGTG
                     ************************************************************
```

Supplemental Fig. 1 (Continued)

```
STRG1648_denovo       TAAATTTATTTTTATTGGTAGATAATTTATTCTCTTTGTTTGACCTTTAATTTGTATAAA
STRG1648_sangar       TAAATTTATTTTTATTGGTAGATAATTTATTCTCTTTGTTTGACCTTTAATTTGTATAAA
Rychc_Li_etal_2022    TAGATTTATTTTTATTGGTAGATAATTTATTCTCTTTGTTTGACCTTTAATTTGTATAAA
                      ** *********************************************************

STRG1648_denovo       GCATTAATTTCTCTTTCTTAATTTGTCATACTTAAACATGGTCTTATAGTAATTTTATTT
STRG1648_sangar       GCATTAATTTCTCTTTCTTAATTTGTCATACTTAAACATGGTCTTATAGTAATTTTATTT
Rychc_Li_etal_2022    GCATTAATTTCTCTTTCTTAATTTGTCATACTTAAACATGGTCTTATAGTAATTTTATTT
                      ************************************************************

STRG1648_denovo       TTACTAAATTTCAGGGTTCTAAAAAAGTAAAAATATTGGTGGTAGAACGACAAGCATTAA
STRG1648_sangar       TTACTAAATTTCAGGGTTCTAAAAAAGTAAAAATATTGGTGGTAGAACGACAAGCATTAA
Rychc_Li_etal_2022    TTACTAAATTTCAGGGTTCTAAAAAAGTAAAAATATTGGTGGTAGAACGACAAGCATTAA
                      ************************************************************

STRG1648_denovo       AGGGTGTGAAGCTAAGCACCAAAGCATTTCAGAAAATGATAAATCTTAAGATTCTTAAAA
STRG1648_sangar       AGGGTGTGAAGCTAAGCACCAAAGCATTTCAGAAAATGATAAATCTTAAGATTCTTAAAA
Rychc_Li_etal_2022    AGGGTGTGAAGCTAAGCACCAAAGCATTTCAGAAAATGATAAATCTTAAGATTCTTAAAA
                      ************************************************************

STRG1648_denovo       TCGACGACTTACATATTAGTGGAGATTTTGAGCTATTGTCCAAGGAGCTCAGATGGCTGT
STRG1648_sangar       TCGACGACTTACATATTAGTGGAGATTTTGAGCTATTGTCCAAGGAGCTCAGATGGCTGT
Rychc_Li_etal_2022    TTGACGACTTACATATTAGTGGAGATTTTGAGCTATTGTCCAAGGAGCTCAGATGGCTGT
                      * **********************************************************

STRG1648_denovo       CTTGGAAAGGATGCCCTTTAAAATGTATACCGTCAAATTTTCCATCTGAGAAACTTGTAT
STRG1648_sangar       CTTGGAAAGGATGCCCTTTAAAATGTATACCGTCAAATTTTCCATCTGAGAAACTTGTAT
Rychc_Li_etal_2022    CTTGGAAAAGATGCCCTTTAAAATGTATACCGTCAAATTTTCCATCTGAGAAACTTGTAT
                      ******** ***************************************************

STRG1648_denovo       TTCTGAATATGAAAGGGAGCAATATCCAAGAATTTGGTTTGAATTTGCAGGTCCGTTACT
STRG1648_sangar       TTCTGAATATGAAAGGGAGCAATATCCAAGAATTTGGTTTGAATTTGCAGGTCCGTTACT
Rychc_Li_etal_2022    TTCTGAATATGAAAGGGAGCAATATCCAAGAATTTGGTTTGAATTTGCAGGTCCGTTACT
                      ************************************************************

STRG1648_denovo       CAATTCTGCAATTTCATGTGTAATGTTTAAGAGAAAGAAAATCTTAATTGAAACAATATT
STRG1648_sangar       CAATTCTGCAATTTCATGTGTAATGTTTAAGAGAAAGAAAATCTTAATTGAAACAATATT
Rychc_Li_etal_2022    CAATTCTGCAATTTTATGTGTAATATTTAAGAGAAAGAAAATCTTAATTGAAACAATATT
                      ************** ********* ***********************************

STRG1648_denovo       TTGTTTACTTCTTAGTATTGTAGAAGTTTGAAGGAGCTGAATCTCTCTGATTGCAAGCGC
STRG1648_sangar       TTGTTTACTTCTTAGTATTGTAGAAGTTTGAAGGAGCTGAATCTCTCTGATTGCAAGCGC
Rychc_Li_etal_2022    TTGTTTACTTCTTAGTATTGTAGAAGTTTGAAGGAGCTGAATCTCTCTGATTGCAAGCGC
                      ************************************************************

STRG1648_denovo       CTCAGAAAAACTCCAAACTTCAACGGTTCACGAAGTCTCAAGACTTTGTGTCTTGAGAAT
STRG1648_sangar       CTCAGAAAAACTCCAAACTTCAACGGTTCACGAAGTCTCAAGACTTTGTGTCTTGAGAAT
Rychc_Li_etal_2022    CTCAGAAAAACTCCAAACTTCAACGGTTCACGAAGTCTCAAGACTTTGTGTCTTGAGAAT
                      ************************************************************

STRG1648_denovo       TGCTCAAGTCTTAAGGAGATCCATCCATCAATAGGAAATTTGGACAGACTAATTCATCTT
STRG1648_sangar       TGCTCAAGTCTTAAGGAGATCCATCCATCAATAGGAAATTTGGACAGACTAATTCATCTT
Rychc_Li_etal_2022    TGCTCAAGTCTTAAGGAGATCCATCCATCAATAGGAAATTTGGACAGACTAATTCATCTT
                      ************************************************************

STRG1648_denovo       CAACTGAATGGTTGCGAAAAGATTACGGATCTTCCGAGCAGCATATGCCAGCTAAAATCC
STRG1648_sangar       CAACTGAATGGTTGCGAAAAGATTACGGATCTTCCGAGCAGCATATGCCAGCTAAAATCC
Rychc_Li_etal_2022    CAACTGAATGGTTGCGAAAAGATTACGGATCTTCCGAGCAGCATATGCCAGCTAAAATCC
                      ************************************************************

STRG1648_denovo       CTTGAAGACTTGTACATTAATGACTGCTCATCTTTACAAACACTGCCAGTTGACATTGGA
STRG1648_sangar       CTTGAAGACTTGTACATTAATGACTGCTCATCTTTACAAACACTGCCAGTTGACATTGGA
Rychc_Li_etal_2022    CTTGAAGACTTGTACATTAATGACTGCTCATCTTTACAAACACTGCCAGTTGACATTGGA
                      ************************************************************

STRG1648_denovo       GATATGCAATGCCTAAGATATCTTAATGCACGTGAAACAGGTATAAAAGAATTGCCTGGA
STRG1648_sangar       GATATGCAATGCCTAAGATATCTTAATGCACGTGAAACAGGTATAAAAGAATTGCCTGGA
Rychc_Li_etal_2022    GATATGCAAAGCCTAAGATATCTTAATGCACGTGAAACAGGTATAAAAGAATTGCCTGGA
                      ********* **************************************************
```

Supplemental Fig. 1 (Continued)

```
STRG1648_denovo          TCTGTTGAAATGCTAGGAAATCTTAGAAATTTGGAAATGGGAGGTCAATACTTAGAGACC
STRG1648_sangar          TCTGTTGAAATGCTAGGAAATCTTAGAAATTTGGAAATGGGAGGTCAATACTTAGAGACC
Rychc_Li_etal_2022       TCTGTTGAAATGCTAGGAAATCTTAGAAATTTGGAAATGGGAAGTCAATACTTAGAGACC
                         ****************************************** *****************

STRG1648_denovo          AAAAGGAGGTTTTCTCAAACAAGAGTACGCCCCATAGTGTCCTTGTCAAAATTTATTTCG
STRG1648_sangar          AAAAGGAGGTTTTCTCAAACAAGAGTACGCCCCATAGTGTCCTTGTCAAAATTTATTTCG
Rychc_Li_etal_2022       AAAAGGAGGTTTTCTCAAACAAGAGTACGCCCCATAGTGTCCTTGTCAAAATTTATTTCG
                         ************************************************************

STRG1648_denovo          GTTTTACGCCTTCCATATTGTGGTTTCTCGGAGGTTGATGTTCCTAGGGATATTGGGAGT
STRG1648_sangar          GTTTTACGCCTTCCATATTGTGGTTTCTCGGAGGTTGATGTTCCTAGGGATATTGGGAGT
Rychc_Li_etal_2022       GTTTTACGCCTTCCATATTGTGGTTTCTCGGAGGTTGATGTTCCTAGGGATATTGGGAGT
                         ************************************************************

STRG1648_denovo          TTATCCAACTTACATCATTTAGATTTGAGCGGCAACAGTTTCCTCTATCTACCCTTTGAT
STRG1648_sangar          TTATCCAACTTACATCATTTAGATTTGAGCGGCAACAGTTTCCTCTATCTACCCTTTGAT
Rychc_Li_etal_2022       TTATCCAACTTACATCATTAGATTTTGAGCGGCAACAGTTTCCTCTATCTACCCTTTGAT
                         *******************    *************************************

STRG1648_denovo          TTTTCCAAGTTACCGTTGTTGAGCTACTTGTTTTTGAATGATTGTGAGAACCTTCAAACA
STRG1648_sangar          TTTTCCAAGTTACCGTTGTTGAGCTACTTGTTTTTGAATGATTGTGAGAACCTTCAAACA
Rychc_Li_etal_2022       TTTTCCAAGTTACCGTTGTTGAGCTACTTGTTTTTGAATGATTGTGAGAACCTTCAAACA
                         ************************************************************

STRG1648_denovo          CTCCCGTCAATATCAAATTTAGAGTACCTTGAAATTCTTGAACTTAGGAATTGCAAAAAA
STRG1648_sangar          CTCCCGTCAATATCAAATTTAGAGTACCTTGAAATTCTTGAACTTAGGAATTGCAAAAAA
Rychc_Li_etal_2022       CTCCCGTCAATATCAAATTTAGAGTACCTTGAAATTCTTGAACTTCGGAATTGCAAAAAA
                         ********************************************* **************

STRG1648_denovo          CTGGTCAAGATTACAGGGTTTGACAACCTCCCTAGTATAAAGATGATCGACATGATCAAT
STRG1648_sangar          CTGGTCAAGATTACAGGGTTTGACAACCTCCCTAGTATAAAGATGATCGACATGATCAAT
Rychc_Li_etal_2022       CTGGTCAAGATTACAGGGTTTGACAACCTCCCTAGTATAAAGATGATCGACATGATTAAT
                         ******************************************************** ***

STRG1648_denovo          TGTACTTCACTGCAGAATCCATTCATTGAAGGCTTCTTTAGTGCCCAGGCTCTATCAATT
STRG1648_sangar          TGTACTTCACTGCAGAATCCATTCATTGAAGGCTTCTTTAGTGCCCAGGCTCTATCAATT
Rychc_Li_etal_2022       TGTACTTCACTGCAGAATCCATTCATTGAAGGCTTCTTTAGTGCCCAGGCTCTATCAATT
                         ************************************************************

STRG1648_denovo          TCATCTAGAAAACATTCAGTGTATGAGGTTAGTCCCTCTCCCTCTCTCTGATCTCTCTCG
STRG1648_sangar          TCATCTAGAAAACATTCAGTGTATGAGGTTAGTCCCTCTCCCTCTCTCTGATCTCTCTCG
Rychc_Li_etal_2022       TCATCTAGAAAACATTCAGTGTATGAGGTTAGTCCCTCTC------TCTGATCTCTCTCG
                         ****************************************      **************

STRG1648_denovo          TATCACATTAATAATTAATGATCTAATTGTGTCTTATTTGTCTCTGATGAAGCTGTTATT
STRG1648_sangar          TATCACATTAATAATTAATGATCTAATTGTGTCTTATTTGTCTCTGATGAAGCTGTTATT
Rychc_Li_etal_2022       TATCACATTAATAATTAATGATCTAATTGTGTCTTATTTGTGTCTGATGAAGCTGTTATT
                         ***************************************** ******************

STRG1648_denovo          ACAAATTTATCTCGAAAGCAATGAGATTCCAGATTGGTGCAGCAATAAAGTAACAGCTCC
STRG1648_sangar          ACAAATTTATCTCGAAAGCAATGAGATTCCAGATTGGTGCAGCAATAAAGTAACAGCTCC
Rychc_Li_etal_2022       ACAAATTTATCTCGAAAGCAATGAGATTCCAGATTGGTGCAGCAATAAAGTAACAGCTCC
                         ************************************************************

STRG1648_denovo          ATCTATCTGTTTGACTATGCCCACAGTACATAATAACAACTTCTTAGGAATGGTTCTCTG
STRG1648_sangar          ATCTATCTGTTTGACTATGCCCACAGTACATAATAACAACTTCTTAGGAATGGTTCTCTG
Rychc_Li_etal_2022       ATCTATCTGTTTGACTATGCCCACAGTACATAATAACAACTTCTTAGGAATGGTTCTCTG
                         ************************************************************

STRG1648_denovo          GTTTGTTTGCCGCCTTTGCGATGTACATGAGCATAAACACTTCATTGTTACTGTTGCCCA
STRG1648_sangar          GTTTGTTTGCCGCCTTTGCGATGTACATGAGCATAAACACTTCATTGTTACTGTTGCCCA
Rychc_Li_etal_2022       GTTTGTTTGCCGCCTTTGCGATGTACATGAGCATAAACACTTCATTGTTACTGTTGCCCA
                         ************************************************************

STRG1648_denovo          TATAAAGCGTTCAGGTTTACCGTGGATTTGGTATTTTGATAGATCTGACAGTCACGAAGT
STRG1648_sangar          TATAAAGCGTTCAGGTTTACCGTGGATTTGGTATTTTGATAGATCTGACAGTCACGAAGT
Rychc_Li_etal_2022       TATAAAGCGTTCAGGTTTACCGTGGATTTGGTATTTTGATACATCTGACAGTCACGAAGT
                         ***************************************** ******************
```

Supplemental Fig. 1 (Continued)

```
STRG1648_denovo        ATCATGTGTATATTACTTCTCTTCCGCAAATGATACACCTTTTGAAGGCCTGAACATCAA
STRG1648_sangar        ATCATGTGTATATTACTTCTCTTCCGCAAATGATACACCTTTTGAAGGCCTGAACATCAA
Rychc_Li_etal_2022     ATCATGTGTATATTACTTCTCTTCCGCAAATGATACACCTTTTGAAGGCCTGAACATCAA
                       ************************************************************

STRG1648_denovo        AGGCGGGGAACAGATAACAGTAGAGGATCGCACTGGCAGAGACGTTGTAAAGAAGATAGG
STRG1648_sangar        AGGCGGGGAACAGATAACAGTAGAGGATCGCACTGGCAGAGACGTTGTAAAGAAGATAGG
Rychc_Li_etal_2022     AGGCGGGGAACAGATAACAGTAGAGGATCGCACTGGCAGAGACCTTGTAAAGAAGATAGG
                       ******************************************* ****************

STRG1648_denovo        GATCCATCTGTTATACTCGGACCAACATGGTAATGTTACATCTTTGCCGGGAGTTGTGGA
STRG1648_sangar        GATCCATCTGTTATACTCGGACCAACATGGTAATGTTACATCTTTGCCGGGAGTTGTGGA
Rychc_Li_etal_2022     GATCCATCTGTTATACTCGGACCAACATGGTAATGTTACATCTTTGCCGGGAGTTGTGGA
                       ************************************************************

STRG1648_denovo        TCATTCTTATACTCCCTCCTACCCACAAAGACTTTTGAGCAGGGCATATCAACTCAAACA
STRG1648_sangar        TCATTCTTATACTCCCTCCTACCCACAAAGACTTTTGAGCAGGGCATATCAACTCAAACA
Rychc_Li_etal_2022     TCATTCTTATACTCCCTCCTACCCACAAAGACTTTC-AGCAGGGCATATCAACTCAAACA
                       ***********************************  ***********************

STRG1648_denovo        ACGACAACATATCCAATGAAATCCTACAAGTGAGGTCCGTATCA----------ACAAGC
STRG1648_sangar        ACGACAACATATCCAATGAAATCCTACAAGTGAGGTCCGTATCA----------ACAAGC
Rychc_Li_etal_2022     ACGACAACATATCCAATGAAATCCTACAAGTGAGGTCCGTATCAGTGGATATCAACAAGC
                       ********************************************          ******

STRG1648_denovo        AAAGTTCTGAGAATGTTTTATGTACCATAGAAAATCTGTTCCATAGAAATAGGCAATGGA
STRG1648_sangar        AAAGTTCTGAGAATGTTTTATGTACCATAGAAAATCTGTTCCATAGAAATAGGCAATGGA
Rychc_Li_etal_2022     AAAGTTCTGAGAATGTTTTATGTACCATAGAAAATCTGTTCCATAGAAATAGGCAATGGA
                       ************************************************************

STRG1648_denovo        CCTGGATCTACCAAATGATGACAATGCCTCTAAAATGTCTTTTTGGAAAATGTTTCTCCG
STRG1648_sangar        CCTGGATCTACCAAATGATGACAATGCCTCTAAAATGTCTTTTTGGAAAATGTTTCTCCG
Rychc_Li_etal_2022     CCTGGATCTACCAAATGATGACAATGCCTCTAAAATGTCTTTTTGGAAAATGTTTCTCCG
                       ************************************************************

STRG1648_denovo        GAGAATAAGTAGGTTTCTTACTTATTTTCTTGTGTTTGTTATGTAAGAAAAAAGTTTGTT
STRG1648_sangar        GAGAATAAGTAGGTTTCTTACTTATTTTCTTGTGTTTGTTATGTAAGAAAAAAGTTTGTT
Rychc_Li_etal_2022     GAGAATAA----------------------------------------------------
                       ********

STRG1648_denovo        CTTAAAGTATTTGTATATTTAGACACTACTATCAATCTGTATTATTCAAAATTCTCATCC
STRG1648_sangar        CTTAAAGTATTTGTATATTTAGACACTACTATCAATCTGTATTATTCAAAATTCTCATCC
Rychc_Li_etal_2022     ------------------------------------------------------------


STRG1648_denovo        AACTACAAGCTGATTTGCTAAAAAGTATTTTTCATTC
STRG1648_sangar        AACTACAAGCTGATTTGCTAAAAAGTATTTTTCATTC
Rychc_Li_etal_2022     -------------------------------------
```

Supplemental Fig. 1

Genomic sequence comparison by Clustal W between STRG1648 from *de novo* assembly (upper) ,STRG1648 from Sanger Sequence (middle) and Ry_{chc} reported in Li *et al*.,(2022) (lower). Start codons are indicated by a red box, and stop codons are indicated by a blue box.

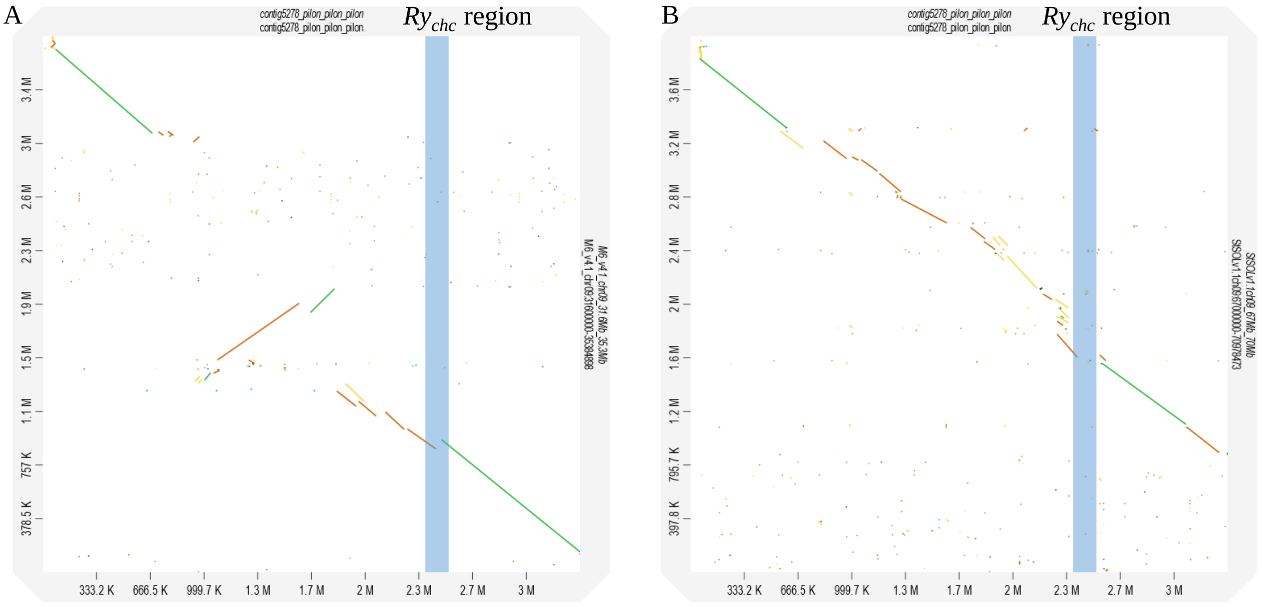

Supplemental Fig.2

a Dot-plot of Contig5278 against chromosome 9 (31.6Mb-35.3Mb region) of *S. chacoense* M6 (Leisner *et al*. 2018).

b Dot-plot of Contig5278 against distal end chromosome 9 (67Mb-70Mb region) of *S. tuberosum* Solyntus (van Lieshout *et al*. 2020). Blue box means Ry_{chc} region on Contig5278.

A

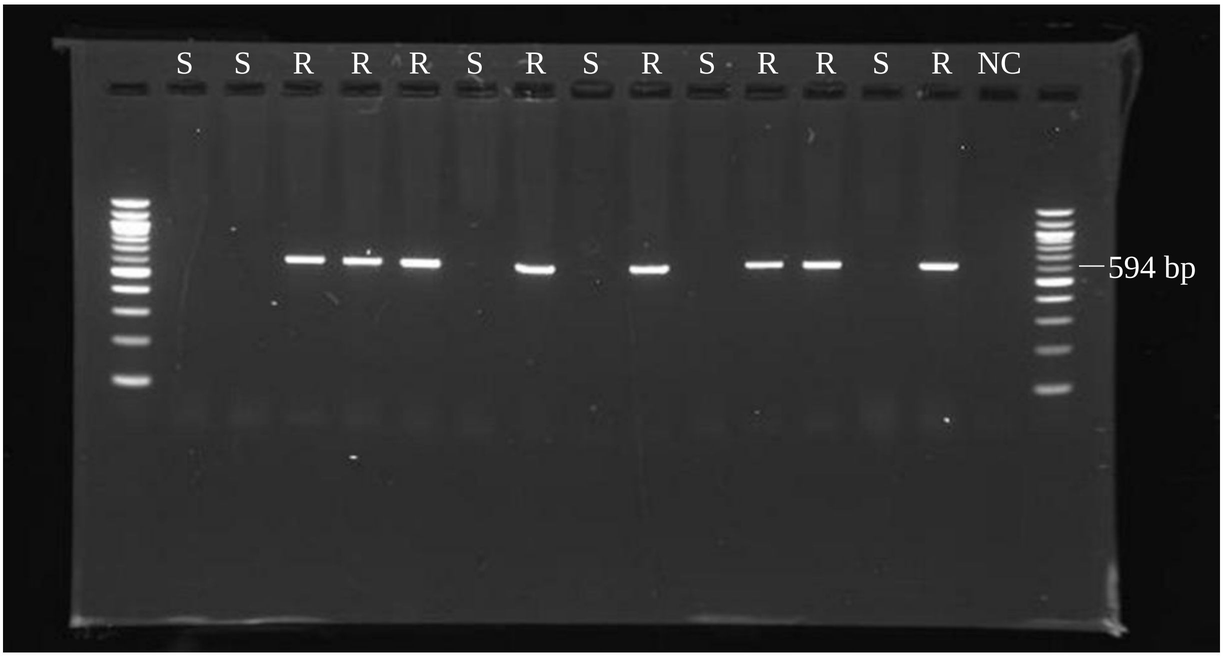

B

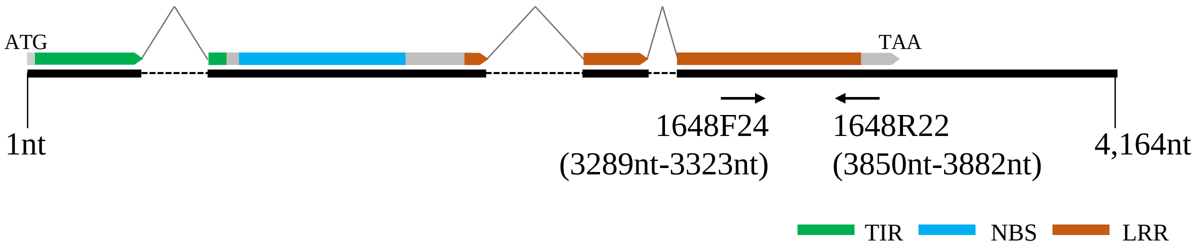

Supplemental Fig. 3

a Overall view of the agarose gel electrophoresis of PCR products by 1648F24/1648R22 primer set in Fig. 4. Lane 1-15: NEB 100 bp DNA Ladder marker; 11023-27(S); 13099-2(S); 13099-5(R); 13099-8(R); 13099-17(R); 13099-22(S); 13099-23(R); 13099-31(S);13096-5(R); 14116-1(S); 14116-5(R); 14116-11(R); Hokkaikogane(S); Sakurafubuki(R); negative control. Electrophoresis was performed in 1x TAE buffer and 3% Agarose 21(Nippongene) gel at 135V for 20 min.

b The locations of 1648F24 and 1648R22 primer set (black arrows). This primer set amplify a 594 bp fragment of the fourth exon (LRR).
