# Supplementary material for: De novo genome assembly of the partial homozygous dihaploid potato identified PVY resistance gene (Rychc) derived from Solanum chacoense
Source: Breed Sci. 2023 Apr 13;73(2):168–79. doi: 10.1270/jsbbs.22078 (PMC10316315; doi:10.1270/jsbbs.22078)
Supplement: Supplementary file 2 — Supplemental Tables [file 73_168_s2.pdf]

doi: 10.1270/jsbbs.22078

Supplemental Table 1 Populations used for fine mapping. Lines/varieties in bold and underlined font carried *Ry*$_{chc}$.

Year	Population name	Female	Male	No. of individuals	No. of recombinants between RY364 and RY186
2013	10080	**Saikai 37**	Kitahime	1,124	4
2013	11023	90101-7	**WB66201-10**	1,104	4
2013	03119	**Konafubuki**	Sayaka	987	3
2013	11165	Hokkaikogane	**Sakurafubuki**	3,926	5
2014	13096	**Konafubuki**	Hokkaikogane	1,884	6
2014	13099	**Sakurafubuki**	Hokkaikogane	4,282	18
2015	14116	**Konafubuki**	Hokkaikogane	5,857	16
2015	14120	**Sakurafubuki**	Hokkaikogane	2,200	6
			total	21,364	62

Supplemental Table 2 List of the PCR primers used in this research.

Primer name	sequence 5'→3'	purpose	Reference
SZRY54-5b	GACTAAGGTTGAAATTGCACTTT	Fine-mapping	this study
SZRY54-5g	CGTGTCAAAGCTATAGAGTTATTACTGC	Fine-mapping	this study
SZRY183-4m	AATGTGATACAAGAGAGAGATCAGAGA	Fine-mapping	this study
SZRY183-4n	CTATGATGTTGATCCTTCTCAAGTG	Fine-mapping	this study
Ry364-14	CTATTATATAAGTCTGGTACTAGGACG	Fine-mapping, qPCR	Takeuchi *et al.* (2008)
Ry364-19	GGCTATATGTTCAATGAATTCATGCTAA	Fine-mapping, qPCR	Takeuchi *et al.* (2008)
RY173-10	CCTAACATTGAACTTCAATGATACA	Fine-mapping	this study
RY173-27	CGTTTCCCATCCGTTGTG	Fine-mapping	this study
RY122-18	GGACTTTCACGCAGAGATG	Fine-mapping	this study
RY122-19	GGCCACAAGATTAAAGACAA	Fine-mapping	this study
RY186-11	TGGTAGGGATATTTTCCTTAGA	Fine-mapping	Takeuchi *et al.* (2008)
RY186-12	GCAAATCCTAGGTTATCAACTCA	Fine-mapping	Takeuchi *et al.* (2008)
SZRY50-2d	ATGATACTTCTCCGTGACTGTTAGG	Fine-mapping	this study
SZRY50-3f	TTTCTCAGTTTCAGAGGTGAAGAT	Fine-mapping	this study
SZRY93-4j	CCAGTTGTTTCACGTCATCT	Fine-mapping	this study
SZRY93-6k	CTGCCAGTGATCTCATTACTCA	Fine-mapping	this study
ASRY22-F	CCAGTTGACATTAGCCTAAC	Fine-mapping	this study
ASRY22-R	AGTTTCATCTCCCACTCGCA	Fine-mapping	this study
ASRY49-F	AAGAGTGTGCATGTGCCAAG	Fine-mapping	this study
ASRY49-R	TGGAGAGCATCTTCTGATGC	Fine-mapping	this study
ASRY63-F	TGGCACTGGCAAAGGTATAG	Fine-mapping	this study
ASRY63-R	AAGTGAGTTGTTTCCGCTCG	Fine-mapping	this study
ASRY76-F	AGGCCCAATGAAATTCCACC	Fine-mapping	this study
ASRY76-R	TCTTGTATAACCTTTTCCTCCATC	Fine-mapping	this study
ASRY91-F	TGCTGCTCAGGCAATTGTTG	Fine-mapping	this study
ASRY91-R	TGATCCACTGTCAGAAGTGG	Fine-mapping	this study
SZRY119-F	CAAAGAGACGCTATATTCACTATACAAT	Fine-mapping	this study
SZRY119-R	CTGCCAGTGATCTCATTACTCA	Fine-mapping	this study
STRG1631f-F	CCGTTGACTCCAACAACTAAAGTAAATAG	sub-cloning	this study
STRG1631f-R	CAGTCGTAGACTCATAGCAATAATAAATTCAAC	sub-cloning	this study
STRG1632f-F	CATAATCAAGAACATCCGAAATTGTCTACAAG	sub-cloning	this study
STRG1632f-R	GCCGTACATAAATATCAAATGTTAATCTCTACACC	sub-cloning	this study
STRG1632f-Fv2	CGAAATTGTCTACAAGCTCCTCTACTCC	sub-cloning	this study
STRG1632f-Rv2	AGCCGTACATAAATATCAAATGTTAATCTCTAC	sub-cloning	this study
STRG1635f-F	CATTATTATTAGTAATTACAAGACTTGGCCGTTG	sub-cloning	this study
STRG1635f-R	GATTTGAGTATTAATTAAGCGTATTTATAAACCG	sub-cloning	this study
STRG1643f-F	GGAACAAACAATTTCTTGTGGGTATATAAATAGC	sub-cloning	this study
STRG1643f-R	ATACTTTTTAGCAAATCTGATTGTAGTTGGATGAG	sub-cloning	this study
STRG1646f-F	GCTGCTTCACATCTCTATTTAATTAATTAAAC	sub-cloning	this study
STRG1646f-R	CAATCAAACCATAGAACCATTCCTAAGAAC	sub-cloning	this study
STRG1648f-F	CTTGTGGATATATAAAGAGCTGCTTCACATCTC	sub-cloning	this study
STRG1648f-R	GAATGAAAAATACTTTTTAGCAAATCAGCTTG	sub-cloning	this study
STRG1649f-F	GTAAATTACTTGACCCAAAATTAAATTTCCAGG	sub-cloning	this study
STRG1649f-R	CTAATTGGTAAAAATTATTCCTATAGCCACCATC	sub-cloning	this study
STRG1650f-F	CTTTGTCCTTCAATTTGTACAAAGCATTTCTC	sub-cloning	this study
STRG1650f-R	GAGATACATACTAGCCGGAGAAGATTTAAGCAC	sub-cloning	this study
aprt F	GAACCGGAGCAGGTGAAGAA	qPCR	Asano and Tamiya (2013)
aprt R	GAAGCAATCCCAGCGATACG	qPCR	Asano and Tamiya (2013)
1648R1	TTTAACCTCTCTTTGCAATCGAGAATTTTG	candidate gene, Sanger sequencing	this study
1648R2	TTCGCACTTGAGAAGGATCAACATCATA	candidate gene, Sanger sequencing	this study
1648R3	ATCCAGATAAATTTGCTGCTTTAGTAAGTGCA	candidate gene, Sanger sequencing	this study
1648R4	AGACTTTACAATGTTCCAAAATTAACCAAC	candidate gene, Sanger sequencing	this study
1648R5	GTTTTATAATACTTTCAATAAACCTTGATTCATG	candidate gene, Sanger sequencing	this study
1648R6	TTAACACCTTCAGCAACACTATCAACTTC	candidate gene, Sanger sequencing	this study
1648R7	CCACCTGAATATTTTATTATGTCTTGTGC	candidate gene, Sanger sequencing	this study
1648R8	TAGAATGGAGATTATTCCTTGTCCCAATCGAC	candidate gene, Sanger sequencing	this study
1648R9	CATCAAACTGTTGAAATATTCGATTATAGATTTC	candidate gene, Sanger sequencing	this study
1648R10	TCTTTCTCTTAAACATTACACATGAAATTGCAG	candidate gene, Sanger sequencing	this study
1648R11	TAACAAACACAAGAAAATAAGTAAGAAACCTAC	candidate gene, Sanger sequencing	this study
1648F11	CATTTCAGAAAATGATAAATCTTAAGATTCTTA	candidate gene, Sanger sequencing	this study
1648F12	AATGTTACATCTTTGCCGGGAGTTGTGGAT	candidate gene, Sanger sequencing	this study
1648F13	TCATGTGTATATTACTTCTCTTCCGCAAATGATAC	candidate gene, Sanger sequencing	this study
1648F14	GCTGTTATTACAAATTTATCTCGAAAGCAATGAG	candidate gene, Sanger sequencing	this study
1648F21	GTTGCCCATATAAAGCGTTCAGGTTTACC	candidate gene, Sanger sequencing	this study
1648F22	GCCTTTGCGATGTACATGAGCATAA	candidate gene, Sanger sequencing	this study
1648F23	ATCTGTTTGACTATGCCCACAGTACATAATAACAAC	candidate gene, Sanger sequencing	this study
1648F24	ACAACCTCCCTAGTATAAAGATGATCGACATGATC	candidate gene, Sanger sequencing	this study
1648F25	TTGATTTTTCCAAGTTACCGTTGTTGAGCTAC	candidate gene, Sanger sequencing	this study
1648F26	CCCATAGTGTCCTTGTCAAAATTTATTTCGG	candidate gene, Sanger sequencing	this study
1648F27	TGGATCTGTTGAAATGCTAGGAAATCTTAGAA	candidate gene, Sanger sequencing	this study
1648F28	GGAAATTTGGACAGACTAATTCATCTTCAAC	candidate gene, Sanger sequencing	this study
1648R21	TCAGATCTATCAAAATACCAAATCCACGGTA	candidate gene, Sanger sequencing	this study
1648R22	GTATAACAGATGGATCCCTATCTTCTTTACAAC	candidate gene, Sanger sequencing	this study
1648R23	TCACAATCATTCAAAAACAAGTAGCTCAACAAC	candidate gene, Sanger sequencing	this study
1648R24	GTAAAACCGAAATAAATTTTGACAAGGACACTATGG	candidate gene, Sanger sequencing	this study

Supplemental Table 3 Results of the PVY inoculation tests of recombinant lines.

		Symptoms (Innoculated leaves/ Upper leaves)[1.2]		ELISA[3]	
Lines/varieties	PVY resistance	PVY^{O}	PVY^{NTN}	PVY^{O}	PVY^{NTN}
13099-2	Susceptible	-/-	m/-	+ (5/6)	+ (4/4)
13099-5	Resistant	-/NS	-/NS	- (0/6)	- (0/5)
13099-8	Resistant	-/NS	-/NS	- (0/5)	- (0/5)
13099-17	Resistant	-/sNS	-/sNS	- (0/5)	- (0/3)
13099-22	Susceptible	(NS)/NS	-/-	+ (2/6)	+ (6/6)
13099-23	Resistant	-/sNS	(NS)/sNS	- (0/6)	- (0/6)
13099-31	Susceptible	-/-	-/-	+ (5/5)	+ (6/6)
13096-5	Resistant	-/NS	-/NS	- (0/5)	- (0/5)
14116-1	Susceptible	-/-	(m)/-	+ (5/5)	+ (5/5)
14116-5	Resistant	-/NS	-/NS	- (0/4)	- (0/4)
Sayaka	Susceptible	m/sNS	m/sNS	+ (2/4)	+ (1/4)
Konafubuki	Resistant	-/NS	-/(VN)	- (0/4)	- (0/4)

[1)] NS: necrosis spot, sNS: small necrosis spot, VN: vein necrosis, m: weak mosaic, -: no symotoms. ( ) means it is infrequent symptoms.

[2)]Symptoms of inoculated leaves are observed seven days after inoculation. Symptoms of upper leaves were observed 21 days after inoculation.

[3)]Lines showing absorbance value more than twice that of non-innoculated plants were judged to be susceptible.

Supplemental Table 4 Results of dihaploid induction to *Ry_{chc}* duplex tetraploid lines.

Female	*Ry_{chc}*	Haploid inducer	No. of berries	No. of true seeds	No.of true seeds without embryo Spot	No. of progenies	No. of clones which have *Ry_{chc}*	No.of clones with homozygous *Ry_{chc}*
13077-7	duplex	*S. phureja* IVP35	16	29	14	9	5	0
13082-3	duplex	*S. phureja* IVP35	17	44	22	7	3	1

Supplemental Table5 The nucleotide sequences of the eight candidate genes.

Gene Name	Genomic sequence	Position on Contig5278 (nt)	Genomic Length (nt)	Transcript Length (nt)
**STRG1631**	ATCCTCAGATTTAGAAAAAAAAAGAAATGGCTGAAATTCTTCTTACAGCAGTCATCAATAAATCTGTAGAAATAGCTGCAAATCTACTCTTTC<br>AACAAGGAACGCGTTTATATTGGTTGAAAGAAGACATCGATTGGCTCCAGAGAGAAATGAAACACATTCAATCATATGTAGACAATGCAAA<br>GGCCAAGGAAGTTGGAGGCGATTCAAGGGTCAAAAACTTATTAAAAGATATTCAACAACTGGCAGGTGATGTGGAAGATCTCTTAGATGAC<br>TTCCTTCCAAAAATTCAACATTCCAACAAGGGCAAAGGTGCTATTTGTTTTCTAAAGGCGGCTTGCATCCCTTGTGCTTCTGTTGCCAATGAGT<br>TTGCTATGGAGATTGAGAAGTTAAGAAGAAGGGTTGCTGACATTGATCGTGTAAGGGCAACTTACAACATCACAGATACAAGTAACAACAA<br>TAATGATTGCATTCCAACGGAACAGAGAAGAAAATTCCTTCATGCTGATGAAACAGAGGTCATCGGTTTGGATGATGACTTCAACAAGCTCC<br>AAGACAAATTGCTTGTTCAAGATTTGTGTAATGGAGTTGTTTCAATAGTTGGCATACCCGGTCTAGGAAAAACAACTCTTGCCAAGAAACTTT<br>ATAGGCATGTCTGTCATCAATTTGAGTGTTCTGCACTGGTCTACGTTTCACAACAGCCAATAGCAGGAGAAATCTTACTTGACATAGCCAAGC<br>AAGTTGGACTGACGGACGAGCAAAGGAAAGAACACTTGGAGAACAATCTAAGATCACTTTTGGAAACAAAAAGGTATGTTATTCTCTTAGA<br>TGACATTTGGGATACTAAATTCTGGGATGCTCTGAACCGTGTCCTTCGTCCTGAATGTGATTCAAAAATTGGCAGTAGGATAATTATCACTTC<br>TCGATATGATCATGTAGGCAGATACATAGGAGAGGATTTCTCGCTCCATGAGTTGCAACCCTTAGATTCAGAGAAAAGTTTTGAACTCTTTAC<br>CAAGAAAATCTTTATTTTGATAATAATAATAATTGGGCTAATGCTTCACCTGTCTTGGTAGATATTGGTAAAAGTATAGTTCGGAGATGTGG<br>AGGTATTCCATTAGCCATTGAGGTGACGGCAGGCATGTTAAGGGCAAGAGAAAGAACGGAACATGCATGGAATAGAGTGCTTGAGCGTATA<br>GGTCATAATATTCAGGATGGATGTGCTAGGGTATTGGCTCTGAGTTACAATGATTTGCCCATTGCATTAAGGCCATGTTTCTTGTATTTTGGCC<br>TTTATCCTGAGGACCATGAAATTCGTGGTTTTGATCTGATAAATATGTGGATTGCTGAGAAGTTGATAGTTGTAAATAGTGGTAATAGGCGAG<br>AGGCTGAAAGTTTGGCGGAGGATATAATAAATGATTTGGTTTCAAGAAACTTGATTCAAGTTGCCAAAAGGACATATGATGGAAGAATTTCA<br>AGTTGTCGCATACATGACTTGTTACATAGTTTGTGTGTTGACTTGGCTAAGGAAAGTAACTTCTTTCACACCGAGCACAATGCATTTGGTGAT<br>CCCGGCAATGTTGCTAGGGTGCGAAGGATTACATTCTACTCTGATACTAATGCCATGAATGAGTTCTTCCGTTCAAATCCTAAGCTTGAGAAG<br>CTTCGTGCACTTTTCTGTTTCACAAAAGACCCTTCCATATTTTCTCAACTGGCTCATCTTGATTTCAAATTATTGCAAGTGTTGGTTGTAGTCAA<br>GCCTCGAAAATTTAATGATTGTATCATTATCCAAAACAAAATTGGGAACATGAGTTGCTTACGCTATCTGCGATTCGAGGGGGGTATTGATG<br>GGAAACTGCCAAATTGTATGGTGAAGCTCAAACATCTAGAGACCCTAGATATTGGTAATAGCCTCATTGATCCTCCTACTGGTGTTTGGAAGT<br>CTACACAATTGAGACATCTTCGTTATAGCGCTTTCAATCAAGTATCTAACTGTTGCAATTCTATAAGCCCAGTTTTTCCAAACGTGCCTCCTAA<br>TAATGTACAAACTTTGATGTGGATGGATGGTGAATTTTTTGAACCGAGATGGTTGCACCGATTTATCAATTTAAGAAAACTGGGTTTAGAGGA<br>AGTATCCGATTCTACCATTAAGAAATTATCAACATTGAGCCCTGTGCCAACGACACTGGAGGTTCTAAAGCTCAGCTCATTTTTCAGTGAATT<br>GAGAGAGCAAATAAACTTGTCGTCGTATCCAAATATTGTTAAGTTGCATTTGAGAGGAAGAATTCTCTTGAACTTTGAAGCATTCCCTCCAAA<br>TCTTGTCAAGCTTACTCTTATCGACTGTAAGGTAGACGATCATGTAGTGGCAGTGCTTAAGAAATTACCCAAATTAAGGATACTTAAAATGGT<br>TTGGTGCAGTTATAATGAAGAAAAGATGGATCTCTCTGGTGATGGTGATAGCTTTCCGCAACTTGAAGTTCTGCATATTCAAGAATCAATTTG<br>GTTGTCTGAAATAACATGCACGGATGATGTGAGTATGCCAAAATTGAAAAAGCTATTACTTATAAGAGTCCATTCACCAATTAGTCTCTCGG<br>AAAGACTTGCAAAGCTGAGAGTATGAAAATCCCAATGTGTCAACATGTTAGTTATTTACTTTTAATATCTCAAAATAAGCTCATTTTTTATTA<br>ATTAATTCATGAACTAAATATTTTATGTCTAATAAATTGCAGATGCTTTTCAGAATGATTATGTCTTTGCTGGAGAGCATCTTCTGATGCCTGT<br>TTGTATTTGTAATAATTAAATTAAATGTTTGATTGCTTCTTCAAGTTGATGTATTTGTGGCTTCTAATTTGTAAAATAAATATATCAACCTTGGC<br>ACATGCGCACTCTTTATG	2,300,618-2,303,512	2,895	2,895
**STRG1632**	TTCCAAAAATGGTGTCTTCAAAATTAGAAGATGTTCTAATTTATAACATAAAATTATCCTCAGTTGGATAACAGGACAAGATGTTGTTTATGA<br>GCCAAGTAACATGGATTTGGCCATGAAATTACTTTATTTTTGATAATAATAATTCAGCTGACTTAGTAAATATTGGTAGAAGTATAGTTGAGA<br>GATGCAGAGGTATACCACTAGCCATTGCGGTGATAGCAGGCATGTTAAGGGCAAAAGAAAGAACAGAATATGCATGGAGCAGAGTGCTTGA<br>GAGTTTGGGTCATAAAATTCAGGATGGATGTGCTAAGGTACTGGCACTGAGTTACAATGATTTGCCCATTGCACTAAGGCCATGTTTCTTGTA<br>CTTTGACCTTTACCCTGAGGACCATGAAATTCATGCTTTTGATTTGACAAATATGTGGATTGCTGAGAAGTTGATAGTTGTAAATAGTGGTAA<br>TATGCAAGAGGCTGAAGATTTGGTGGAGGATGTCCTTAATGATTTGGTTTCTAGAAACTTGATTCAAGTTGCCAAATGGAAATATAATGGAA<br>GAATTTTAAGTTGTCGCATACATGACTTGTTACATAGTTTGTGTGTGGACTTGGCTGAGGAAAGTACGTAACTTCTTTCACACTGAGCACAAT<br>GCATTTGGTGATCTCCGCAATGTTGCTAGGGTGCGAAGGATTACATTCTACTCTGATAATGTAATGAATGAGTTCTTTCGTTCAAATCCTAATC<br>CAAAGAAGCTTCTTGCACTTTTCTGTTTCACAAAAGACCGTTGCATATTTTCTCAATTGGCTCATCTTGACTTCAAATTATTGCAAGTGTTGGT<br>TGTAGTTATGTCTCCAGATGGTTATGGGGGTTTAACTATGAGAAACAAATTTGGGAAGATGAGTTGCTTATGCTATCTGCGATTGGAGAGGG<br>AATTAGAGGAAAATTGCCAAATAATATTGTGAAGCTCAAATGTCTAGAGACCATAGATATTGGAGTGGGCGACATTGAACTTCCTTGTAGTG<br>TTTGGGATTTTAAACAATTGAGACATCTTCATTGTAGAGAAGAACTTAAGGGTTTCTTTTCTATAAGCCCAAACATGTACTCATTGGCTCATA<br>ATAATCTACAAACTTTGATGTGGATGGTAAATTTTTTGAGACGAGATTGTTGCACCAATTGATAAATTTAAGAAAACTGGGTATAAGTTCAGC<br>ATCTGATTCTACCATTAATATATTATCAGCATAGAGCCATGTGCCAACAACGTTGGAGGTTCTGAAGCTCAAATTTTGCAATGAATCGAGTGA<br>GCAAACAAACTTGTCGTCCTATCCAAATATTGTTAAGTTGCATTTGGAAAGAAAAATGCCGTTGAACTGTGAAGCATTCCCTCCAAATCTGGT<br>CAAGCTTACTCTTGTCTACTTGACGGTAGATGATCATGTAGTGGCAGTGCTTAAGAAATTGCTCAAATTACGAATACTTAAAATGGTTGTCTG<br>CGAACATAAAAAAGAAGAGATGGATCTCTCTGGTGATGGCTTTCCGCAACTTGAAGTTTTGCATATTCAATATCCAATTTGGTTGCCTGGAAT<br>AACATGTATGGATGATGTCAGTATGCCTAAACTGAACAAGCTATTTCTTGTAGATACCAAATGCGAGAGCTCAGTACGTCTTTCCGAACGTCT<br>TGCAAAGCTGAGAGTATGAAAATCCCAAGCTTATTTACTTTTATATCTAATAAATTGCAGATGCATTTCAGAATATGAGTATGTTTGTATTTGT<br>AATTAAATAAATAAAATG	2,310,970-2,312,752	1,783	1,783
**STRG1635**	CTTATCCTTTTACCAATAATTGATCATCTTTTTCTCTTACTTTCCCTTGCTAATCCTCACATTTAGAAAAAAGAAATTCAAGGAAAAAAGAGAT<br>GGCTGAAATTCTTCTCACAGCAGTCATCAATAAATCTGTAGAAATAGCTGGAAATGTACTCTTTCAAGAAGGAACGCGTTTATATTGGTTGAA<br>AGAGGACATAGATTGGCTCCAGAGAGAAATGAGACACATTCGATGATATGTAGACGATGCAAAGGCCAAGGAAGTTGGAGGCGATTCAAG<br>GGTCAAAAACTTATTAAAAGATATTCAACAACTGGCAGGTGATGTGGAGGATCTCTTAGATGAGTTTCTTCCAAAAATTCAACAATCCAACA<br>AGTTCAAAGGCGCAATTTGTTGCCTTAAGACGGTTTCTTTTGCGGATGAGTTTGCTATGGAGATTGAGAAGATAAAAAGAAGAGTTGCTGAC<br>ATTGACCGTGTAAGGACAACTTACAACATCATGGATACAAGTAATAACAATAACGATTGCATTCCATTGGACTAAAGAAAATTGTTCCTTCA<br>TGCTGATGAAACAGAGGTCATCGGTTTGGATGATGACTTCAATATGCTACAAGCCAAATTACTTGATCATAATTTGCCTTGTGGACTTGTTTC<br>CATAGTTGGCATGCCCGGTCTAGGAAAAACAACTCTTGCCAAGAAACTTTATAGGCAAGTCCGTGATAAATTTGAGTGTTCGGGACTAGTCT<br>ACATTTCACAACAGCCAAGAGCAGGAGAAATCTTACATGACATAGCCAAACAAGTTGGAGTGACGGAAGAGGAAAGGAAAGATAACTTGG<br>AGGGCAAACTAAGATCACTGTTGAAAAGAAAAAGGTATGTTATCCTCCTAGATGATATTTGGGATGTTGAAATTTGGAACTATCTGAAACTT<br>GTCCTTCCTGAGTGTGATTCAAAAATTGGCAGTAGGATAATAATCACATCTCGAAATAGTAATGTAGGAAGATACATAGGAGGAGATACCTC<br>ACTCCACTTGTTGCAACCCCTAGATTCAGAGAACAGTTTTGAACTCTTTACCAAGAAAATCTTTACTTTTGATAACAATAATAATTGGGTCAA<br>TTCTTCACCAAACTTGGTAGATATTGGTAGAAGTATAGTTGGTAGATGCGGAGGTATACCTCTAGCGATTGTGGTAACTGCAGGCATGCTAA<br>GGGCAAGAGAAAGAATAGAACATACATGGAACAGAGTACTTGAGAGTATGGGTCATAAAATTCAAGATGGATGTGGTAACGTATTGGCTCA<br>GAGTTACAATGATTTGCCCATTGCATTAAGGCCATGTTTCTTGTACTTTGGCCTATTCCCCGAGGACCATGAAATTCGTGCTTTTGATTTGACA<br>AATATGTGGATTGCTGAGAAGCTGATAGTTGTAAATAGCGGCACTATGCGAGAGGCTGAAGATTTGGCGGAGGATGTCCTAAATGATTTGGT<br>TTCTAGAAACTTGATTCAAGTTGCCAAAAGGACATATGATGGAAGAATTTCAAGTTGTCGCATACATGACTTATTACATAGTTTATGTGTGGA<br>CTTGGGTAAGGAAAGTAACTTCTTTCACACCGAGCACAATGCATTGGGTGATCTCCGCAATGTTTCTAGGGTGCGAAGGATTACATTCTACTC<br>CGATAATAATGCCATGAGTAAGTTCTTCCGTTCAAATCCTAAGCCCAAGAAACGTGCACTTTTGTGTTTCACAAAAGACCGTTGCATATTTTC<br>TGAATTGGCTCATCTTAACTTCAAATTATTGCGAGTGTTTGTTGTAATCATGTCTCAAGATGGTTATGGGGGTTTCACTATGGGAAACAGATTT<br>GGGAAGATGAGTTGCTTACGCTATCTGCAATTGGAGGGGGTAATTAGAGGAAAATTGCCAAATAATATTGTGAAGCTCAAATGTCTAGAGAC<br>CATAGATATTGGAGTGGGCGACATTGAACTTCCTTGTAGTGTTTGGGATTTTAAACAATTGAGACATGTTTATTGTAGAGAAGAACTTAAGGG<br>TTTCTTTTCTATAAGCCCAAACATGTACTCATTTCATGATAATGTAGAAACTTTGATGTGGATGGATGATAAATTTTTTGAGGCGATATTGTTG<br>CACCAATTGATAAATTTAAGAACACTGGGTGTAAGTTCAGCATCTGATTCTACCATTAAGATGTTATCAGCATTGAGCCCTGTGCCAACAGCG<br>TTGGAGGTTCTGAAGCTCAAATTTTGCAATGAATCGAGTGAGCAAATAAACTTGTCGTCCTATCCAAATATTGTTAAGTTGCATTTGGAAAGA<br>AGAATGCCGTTGAACTGTGAAGCATTCCCTCCAAATCTTGTCAAGCTTACTCTTGTCTACTTGATGGTAGACGCTCATATAGTGGCAGTGCTT<br>AAGATATCGCCCAAAGTACAAATACTTAAAATGGTTGGCTGCGAACATAAAAAAGAAGAGATGGATCTCTCTGGTGATGGCTTTCCGCAACT<br>TGAAGTTTTGCATATTCAAAATCCACTCTGGTTGCCTAAAATAACATGTACGGATTATGTCAGTATGCCTAAACTAAACAAGCTATTACTTGT<br>AGAGACCAAATGCGAGAGGTCAGTATGTCTCTCCAAACGTCTTGCAAAGCTGAGAATATGAAAATCTCGATGTGTCTACAGATGCATTTCAA<br>AATGATTTAAGTCTTTGCTGGAGAACATCTTCAATGTCTTCTGATGCATGTTTGTATTTGTAATAAAAAGTTTGATTGCTTTCTGAAGTTGATA<br>TATTTGTGGCTTGTGATTTGTAAAACATATTTTATTTATTGTCTTATGTATATTTACCTTTGGAATTAGCGGTAGCTTTCGTTTCTTTTCTTCTTC<br>AATAAACAATGCTCGCTTACGGGTGTGCATCTCTCGGTTCGATTT	2,324,216-2,327,231	3,016	2,917
**STRG1643**	CCCTTTAATTTATTGTCATCAAATATGAATACTCAGTTTGTTGTAGGAGAATCATCTTCTTCTTCCTTCTTGTCTTATGATGTATTTCTCAGTTTC<br>AGAGGTGAAGATACTCGCAAAACCTTCACTGGTCATCTTTACACCTAATTGTGTCAAGTCGGAGCTCATACCTTCATAGATGATGAGGAATT<br>GAGAAAGGGAGACGTCATTTCAATTGAACTTGAGAAAGCAATTGAACAATCTAGGGTTGTCATTGTTGTTTTCTCGAAAAATTATGCTTCCTC<br>TAGTTGGTGTCTTGATGAACTTGTCAAAATTCTCGATTGCAAAGAGAGGTTAAATCAGATAATTTTGCGTATTTTCTATGATGTTGATCCTACT<br>CAAGTGCGAAGGCAAACTGGATCCTTTGGTGAAGCTTTGGCAAAACACAAGGAACGATTGGTTGGAGCAGAAAGAATGGAAAAGTGGAAA<br>GCTGCACTTACTGAAGCAGCAAATTTATCTGGATGGGATTTGACAAATGTTGCTGATGGGTAAGACTTCTTAATTCAACTATTAAATTTTGAA<br>TTTAAGCATTTGTCAACATTTATTTCGTTTTTATGCCTTCCATATTGGGGTTTCTCGGAGGCTGATGTTCCTAGGTATATTGGGAGTTTATCCAA<br>CTTATATTATTTAGATTTGAGCGACAACAGTTTCCTCTATCTATCCTTTGATTTTTCCAAGTTACCATTGTTGATGTCCTTGTGTTTGAATGATT<br>GTGAGAACCTTCAAACACTCCCGTCAATATCAAATTTAAAGTACCTTAGAAATCTTGAACTTTGGAGATTTTGAGCTATTGTCCAAGGAGCTC<br>AGATGGCTGTCTTGGAAAGGATGTCCTTTAAAATGTATACCATCAAATTTTCCATCTGAGAAACTTGTATTTCTGAATATGAAAGGGAGCAAT<br>ATCCAAGAATTTGGTTTGAGTTTGCAGGTTCGTTACTCAATTCTGCAATTTTAGGTGTAATGTTTAAGAGAAAGAAAATCTTAATTGAAACAA<br>TATTTTGTTTACTTCTTAGTATTGTAGAAGTTTGAAGGAGCTGAATCTCTCTGATTGCAAGCACCTCAGAAAAACTCCAAACTTCAACGGTTC<br>ACGAAGTCTCGAGACTTTGTGGCTTGAGAATTGCTCAAGTCTGAAGGAGATCCATCCATCAATAGGAAATTTGGACAGACTAACTTATCTTC<br>AACTGAATGGTTGCGAAAAGATTACGGAACTTCCGAGCAGCATATGCCAGCTAAAATCCCTTAGAGTTTTGTACATTAATTACTGCTCATTTT<br>TACAAACACTGCCAATTGACATTGGAGATATGCAAAGCCTAAGACTTCTTCGTGCACGTCATACAGGTATAAAGCAATTGCCTGGATCTGTT<br>GAAATATTAAGAAATCTTATGAGATTGGATGTGAGAGGTCGGAATTTAGAGGCCAAAAGGAGGTTTTCTCAAACAAGAGTACGCTCCATAGT<br>ATCCTTGTCAAAATTTATTTCGATTCTACGCCCTTCATATTGTGGTTTTGATGTTCCTAGGGATATTGGGAAATTATCCATGTTATTTTGTTTAG<br>ATTTGAGCGGCAACAGTTTCCTCTATCTACCCTTTGATTTTTCCAAGTTACCATTGTTGATGTCCTTGTGTTTGAATGATTGTGAGAATCTTCAA<br>ACACTCCCGTCAATAACAAATTTAGAGTACCTTACAACTCTTGAACTTAGGAATTGCCAAAAACTGGTCGAGATTACAGGATTGGACAACCT<br>CCCTTCAATATATGAGATCGACATGATTAATTGTACTTCACTGCAGAATCCATTCAGTGAATGCTTCTTTAGGGCCCCTGCTCTAGAAAACAT<br>CTACTGTATGAGGTTAGTCCCTCTCCCTCTCTGATCTCTCTCTTGTATCACATTAATAATTAATGATCTAATTAATCTGGAAAGCAATGAGATT<br>CCAGATTGGTGCAGCAATAAAGTAAAAGCTCCATCTGTCTGTTTGACTATGCCCACAGTACATAATAACAAGTTCTTAGGAATGGTTCTATGG<br>TTTGATTGCCACTTTGGCAATCTAACCACCCGTCTTGAAGAGTTTGATGTTACTATTGGCCAGCCTTCAGCTTTTTCCGGACTTGGGTACCTG<br>ACAGTCAGGGAGAAGTATCATGTGTATATTACTTATCTTTCTCAAATGGTAGACCTTTTAATGGCCTGAACATCATCAAAGGCGGGGAACAG<br>ATAAGAGTAGAGGATCGCACTGGCGGAGCCGTAGTAAAGAAGATAGGAGTACATCTATTATATTTAGATCAAAATGGTAATGTTACTTCTTT<br>CCCGGCAGTTGTGGATCATTCTTATACTCCCTCCTATCCACAAAGACTTTCAGCAGGGCATATCAACTCAAACAACAACACATCCAGTGAAA<br>TCTTACAAGTGAGGTCCGTATCAGGGGATATCAATGAGCAAAGTTCTGAGAGTGTGTTCAGTACCATGGAAAATGTGTTCCATAGAAATAGG<br>TTATGGACCTGGATCTACCAAATGATAACAGTGCCTCTAAAATGTCTTTTTAGAAAATGTTTTTCCGGAGAATAATTAGATTTCTTACTTATTT<br>TCTTGTGTTCGTTACGTAAGAAAAAAGTTTGTTCTTAAAGTATTTGTATATTTAGACACTACTATCAATC	2397081-2399757	2,677	2,677

Supplemental Table5 The nucleotide sequences of the eight candidate genes. (Continued)

Gene Name	Genomic sequence	Position on Contig5278 (nt)	Genomic Length (nt)	Transcript Length (nt)
**STRG1646**	GTTGTAGGAGAATCATCTTCTTCTTCCTTCTTGTCTTATGATGTATTTCTCAGTTTCAGAGGTGAAGATACTCGCAAAACCTTCACTGGTCATCT<br>TTACACCTAATTGTGTCAAGTCGGAGTTCATACCTTCATAGATGATGAGGAGTTGAGAAAGGGAGACGTCATTTCAATTGAACTTGAGAAAGC<br>AATTGAACAATCTAGGGTTGTCATTGTTGTTTTCTCGAAAAATTATGCTTCGTCTAGTTGGTGTCTTGATGAACTTGTCAAAATTCTCGATTGCA<br>AAGAGAGGTTAAATCAGATAATTTTGCGTATTTTCTATGATGTTGATCCTACTCAAGTGCGAAGGCAAACTGGATCCTTTGGTGAAGCTTTGG<br>CAAAACACAAGGAACGATTGGTTGGAGCAGAAAGAATGGAAAAGTGGAAAGCTGCACTTACTGAAGCAGCAAATTTATCTGGATGGGATTT<br>GACAAATGTTGCTGATGGGTAAGACTTCTTAATTCAACTATTAAATTTTGAATTTAAGCATTTGTCAACATTTATTTCGTTTTTATGCCTTCCAT<br>ATTGTGGTTTCTCGGAGGCTGATGTTCCTAGGTATATTGGGAGTTTATCCAACTTATATTATTTAGATTTGAGCGACAACAGTTTCCTCTATCTA<br>TCCTTTGATTTTTCCAAGTTACCATTGTTGATGTCCTTGTGTTTGAATGATTGTGAGAACCTTCAAACACTCCCGTCAATATCAAATTTAAAGTA<br>CCTTAGAAATCTTGAACTTTGGAGATTTTGAGCTATTGTCCAAGGAGCTCAGATGGCTGTCTTGGAAAGGATGTCCTTTAAAATGTATACCATC<br>AAATTTTCCATCTGAGAAACTTGTATTTCTGAATATGAAAGGGAGCAATATCCAAGAATTTGGTTTGAATTTGCAGGTTCGTTACTCAATTCTG<br>CAATTTTAGGTGTAATGTTTAAGAGAAAGAAAATCTTAATTGAAACAATATTTTGTGTACTTCTTAGTATTGTAGAAGTTTGAAGGAGCTGAAT<br>CTCTCTGATTGCAAGCACCTCAGAAAAACTCCAAACTTCAACGGTTCACGAAGTCTCGAGACTTTGTGGCTTGAGAATTGCTCAAGTCTGAAG<br>GAGATCCATCCATCAATAGGAAATTTGGACAGACTAACTTATCTTCAACTGAATGGTTGCGAAAAGATTACGGAACTTCCGAGCAGCATATG<br>CCAGCTAAAATCCCTTAGAGTTTTGTACATTAATTACTGCTCATTTTTACAAACACTGCCAATTGACATTGGAGATATGCAAAGCCTAAGACTT<br>CTTCGTGCACGTCATACAGGTATAAAGCAATTGCCTGGATCTGTTGAAATATTAAGAAATCTTATGAGATTGGATGTGAGAGGTCGGAATTTA<br>GAGGCCAAAAGGAGGTTTTCTCAAACAAGAGTACGCTCCATAGTATCCTTGTCAAAATTTATTTCGATTCTACGCCCTTCATATTGTGGTTTTG<br>ATGTTCCTAGGGATATTGGGAAATTATCCATGTTATTTTGTTTAGATTTGAGCGGCAACAGTTTCCTCTATCTACCCTTTGATTTTTCCAAGTTA<br>CCATTGTTGATGTCCTTGTGTTTGAATGATTGTGAGAATCTTCAAACACTCCCGTCAATAACAAATTTAGAGTACCTTACAACTCTTGAACTTA<br>GGAATTGCCAAAAACTGGTCGAGATTACAGGATTGGACAACCTCCCTTCAATATATGAGATCGACATGATTAATTGTACTTCACTGCAGAATC<br>CATTCAGTGAATGCTTCTTTAGGGCCCCTGCTCTAGAAAACATCTACTGTATGAGGTTAGTCCCTCTCCCTCTCTGATCTCTCTCTTGTATCACA<br>TTAATAATTAATGATCTAATTAATCTGGAAAGCAATGAGATTCCAGATTGGTGCAGCAATAAAGTAAAAGCTCCATCTGTCTGTTTGACTATG<br>CCCACAGTACATAATAACAAGTTCTTAGGAATGGTTCTATGGTTTGATTGCCACTTTGGCAATCTAACCACCC	2,423,296-<br>2,425,338	2,043	2,043
**STRG1648**	CATCAAATATGAATACTCAAGGAGAATCATCTTCTTCTTCCAACTTATGTTATGATGTGTTTCTCAGTTTCAGAGGTGAAGATACTCGCAAAAA<br>CTTCATTGATCATCTTTATTTCCGATTATGTCAAGTCGGAGTTAATACTTTCATAGATGATGAGGAATTGAGAAAGGGAGACGTCATTTCAAAC<br>AAACTTGACAAAGCAATTGAACAATCTAGAATTGCCATTGTTGTTTTCTCGAAAAATTATGCTTCGTCTAGTTGGTGTCTTGATGAACTTGTCA<br>AAATTCTCGATTGCAAAGAGAGGTTAAATCAGGTAGTTTTGCCTATTTTCTATGATGTTGATCCTTCTCAAGTGCGAAGGCAAACTGGATCCTT<br>TGACGAAGCTTTGGAAAAACACCAGGAACGATTGATTGGAGCTGAAAGAATGGAAAAGTGGAAAGCTGCACTTACTAAAGCAGCAAATTTA<br>TCTGGATGGGATTTGAGAAATGTTGCTGATGGGCATGAATCAAGGTTTATTGAAAGTATTATAAAACAAGTTCTGCAAGAGGTTAACCAGAC<br>ACCTCTAGATGTTGCTCATTACCCAATTGGATTAGATTCTCCTATCAAACATATAGAGGTGTTACTGCAAAGTGGATGTGAGCATGAAGTTCG<br>CATGGTTGGTATATGTGGCATTGGTGGAATTGGAAAAACAACTTTGGCAAAAGAAATCTATAATCGAATATTTCAACAGTTTGATGGTAGTTG<br>CTTCCTTTCTGACATTAGATCAAAAACTGAAGAATCGGGTCTAATCAAGCTTCAAGAGAAACTACTTTATCAAATCCTCAAAACTAAGGAATT<br>TGAAGTTGATAGTGTTGCTGAAGGTGTTAATCTCATCAAAGCAAGACTTGGGTCTCAGAAGGTTCTAATTGTTCTTGATGACGTGGATCATAG<br>AAGCCAATTAGAATCCTTAACAAGAGAAAGAAGTTGGTTTGGCTTAGGTAGTGTAATAATTATTACAACCCGAGATGAACATTTGCTATATGG<br>GCTTACAACAAGTGAGATATACCAGGCCAAACTTTTAAATGACAAGGAAGCCCAACAACTTTTTTCTTGTCATGCTTTTAACTGTTTTTCTCCA<br>CCACAAGAATATGTTGAACTGGCACAAGACATAATAAAATATTCAGGTGGGCTACCATTAGCTCTTGTGACATTGGGGTCACATCTGCAAGG<br>GAGATCCGTTGAAGAATGGAGATACGAGTTCAAAAAACTAAAAGCAATTCCTCATGGTGATATTCAAAAGATTCTCAAGATAAGCTTTGATG<br>GACTTGACGCCAATACTCAGAGTGTTTTCCTTGATATCGCATTTGCCTTCCATGGTTGTGATGAGGATGAAGTTACCAAAACATTAAATGCGT<br>GTGGTTTTTATTCTGAAAGTGCAATTTCAACCTTAGTACAAAGGAACTTGGTCCAAAGGAATAGGCCTCGTTTGGTGATGCATGATCTAGTGC<br>AGGAAATGGGAAGAGAAATCGTTCGCATGGAATCTCAAGACCCTGGAAAACGGAGTAGATTGTTCAACCCTCAAGAAGTCATTGATGTTCTA<br>CAAGGAAATAAAGGTTCTAAAAAAGTAAAAATATTGGTGGTAGAACGACAAGCATTAAAGGGTGTGAAGCTAAGCACCAAAGCATTTCAGA<br>AAATGATAAATCTTAAGATTCTTAAAATCGACGACTTACATATTAGTGGAGATTTTGAGCTATTGTCCAAGGAGCTCAGATGGCTGTCTTGGA<br>AAGGATGCCCTTTAAAATGTATACCGTCAAATTTTCCATCTGAGAAACTTGTATTTCTGAATATGAAAGGGAGCAATATCCAAGAATTTGGTT<br>TGAATTTGCAGTATTGTAGAAGTTTGAAGGAGCTGAATCTCTCTGATTGCAAGCGCCTCAGAAAAACTCCAAACTTCAACGGTTCACGAAGTC<br>TCAAGACTTTGTGTCTTGAGAATTGCTCAAGTCTTAAGGAGATCCATCCATCAATAGGAAATTTGGACAGACTAATTCATCTTCAACTGAATG<br>GTTGCGAAAAGATTACGGATCTTCCGAGCAGCATATGCCAGCTAAAATCCCTTGAAGACTTGTACATTAATGACTGCTCATCTTTACAAACAC<br>TGCCAGTTGACATTGGAGATATGCAATGCCTAAGATATCTTAATGCACGTGAAACAGGTATAAAAGAATTGCCTGGATCTGTTGAAATGCTAG<br>GAAATCTTAGAAATTTGGAAATGGGAGGTCAATACTTAGAGACCAAAAGGAGGTTTTCTCAAACAAGAGTACGCCCCATAGTGTCCTTGTCA<br>AAATTTATTTCGGTTTTACGCCTTCCATATTGTGGTTTCTCGGAGGTTGATGTTCCTAGGGATATTGGGAGTTTATCCAACTTACATCATTTAGA<br>TTTGAGCGGCAACAGTTTCCTCTATCTACCCTTTGATTTTTCCAAGTTACCGTTGTTGAGCTACTTGTTTTTGAATGATTGTGAGAACCTTCAAA<br>CACTCCCGTCAATATCAAATTTAGAGTACCTTGAAATTCTTGAACTTAGGAATTGCAAAAAACTGGTCAAGATTACAGGGTTTGACAACCTCC<br>CTAGTATAAAGATGATCGACATGATCAATTGTACTTCACTGCAGAATCCATTCATTGAAGGCTTCTTTAGTGCCCAGGCTCTATCAATTTCATC<br>TAGAAAACATTCAGTGTATGAGGTTAGTCCCTCTCCCTCTCTCTGATCTCTCTCGTATCACATTAATAATTAATGATCTAATTGTGTCTTATTTG<br>TCTCTGATGAAGCTGTTATTACAAATTTATCTCGAAAGCAATGAGATTCCAGATTGGTGCAGCAATAAAGTAACAGCTCCATCTATCTGTTTG<br>ACTATGCCCACAGTACATAATAACAACTTCTTAGGAATGGTTCTCTGGTTTGTTTGCCGCCTTTGCGATGTACATGAGCATAAACACTTCATTG<br>TTACTGTTGCCCATATAAAGCGTTCAGGTTTACCGTGGATTTGGTATTTTGATAGATCTGACAGTCACGAAGTATCATGTGTATATTACTTCTCT<br>TCCGCAAATGATACACCTTTTGAAGGCCTGAACATCAAAGGCGGGGAACAGATAACAGTAGAGGATCGCACTGGCAGAGACGTTGTAAAGA<br>AGATAGGGATCCATCTGTTATACTCGGACCAACATGGTAATGTTACATCTTTGCCGGGAGTTGTGGATCATTCTTATACTCCCTCCTACCCACA<br>AAGACTTTTGAGCAGGGCATATCAACTCAAACAACGACAACATATCCAATGAAATCCTACAAGTGAGGTCCGTATCAACAAGCAAAGTTCTG<br>AGAATGTTTTATGTACCATAGAAAATCTGTTCCATAGAAATAGGCAATGGACCTGGATCTACCAAATGATGACAATGCCTCTAAAATGTCTTT<br>TTGGAAAATGTTTCTCCGGAGAATAAGTAGGTTTCTTACTTATTTTCTTGTGTTTGTTATGTAAGAAAAAAGTTTGTTCTTAAAGTATTTGTATA<br>TTTAGACACTACTATCAATCTGTATTATTCAAAATTCTCATCCAACTACAAGC	2,476,328-<br>2,480,621	4,294	3,593<br>4,047
**STRG1649**	AATTAATTAAACAAAAAATTAACTAAAAATCTCTTTAATTTATTGTCATCAAATATGAATACTCAATTTGTTGTAGGAGAATCATCTTCTTCTT<br>CCAACTTATCTTATGATGTGTTTCTTAGTTTCAGAGGTGAAGATACTCGCAAAAACTTCATAGGTCATCTTTATTTCCGATTGTGTCAAGTCGG<br>GGTTAATACTTTCATAGATGATGAGGAATTGAGAAAGGGAGACGTCATTTCAAACAAACTTGACAAAGCAATTGAACAATCTAGAATTGCCA<br>TTGTTGTTTTCTCGAAAAATTATGCTTCGTCTAGTTGGTGTCTTGATGAACTTGTCAAAATTCTCGATTGCAAAGAGAGGTTAAATCAGGTAGT<br>TTTGCCTATTTTCTATGATGTTGATCCTTCTCAAGTGCGAAGGCAAACTGGATCCTTTGACGAAGCTTTGGAAAAACACCAGGAACGATTGAT<br>TGGAGCTGAAAGAATGGAAAAGTGGAAAGCTGCACTTACTAAAGCAGCAAATTTATCTGGATGGGATTTGAGAAATGTTGTTGATGGGCATG<br>AAGCAAAATTTATTGAAAGTATTATAAAACAAGTTCTGCAAGTCGTTAAGCAGACACCTCTAGATGTTGCTCATTACCCAATTGGATTAGATT<br>CTCCTATCAGACATATAGAGATGTTACTGCAAAGTGGATGTGAGCATGAAGTTCGCATGGTTGGTATATGTGGCGTTGGTGGAATTGGAAAA<br>ACAACTTTGGCAAAAGCTATCTATAATAAATTATTTCAACAGTTTGATGGTAGTTGCTTCCTTTCTGACATTAGATCGAAAACTGAAGAATCG<br>GGTCTAGTCAAGCTTCAAAAGAAACTACTCACTCAAATACTCAAAACTAAGGAATTTGAAGTTGATAGTGTTGCTGAAGGTGTTAATCTCATC<br>AAAGCAAGACTTGGGTCTCAGAAGTTTCTAATTGTTCTTGATGATGTGGATCATAGAAGCCAATTAGAATCCTTAACAAGAGAAAGAAGTTG<br>GTTTGGCTCTGGTAGTGTAATAATTATTACAACCCGAGATGAACATTTGCTATATGGGCTTACAACAAGTGAGATATATCGGGCCAAACTTTT<br>AAATGACAATGAAGCCCAACAACTTTTTTCTTGTCATGCATTTAATTGTTTTTCTCCACCACAAGAATATGTTGAACTGGCACAAGACATAATA<br>AAATATTCAGGTGGGCTACCGTTAGCTCTTGTGACATTGGGGTCACATTTGCAAGGGAGATCCATTAAAGAATGGATATACGAGTTCAAAAA<br>ACTAAAAGCAATTCCTCACAGTGATATTCAAAAGATTCTCAAGATAAGCTTTGATGGACTTGATGTCAATACTCAAAGTGTTTTCCTTGATATC<br>GCATGTGCCTTCAATGGTTGTTATGAGAGTGAAGTTACCGAAACATTAAATGCGTGTGGTTTTTATTCTGAAAGTGCAATTTCAACCTTAGTTC<br>AAAGGAACTTGCTCCAAAGGGTTTGTCCTAGTTTGGTTATGCATGGTCTAGTGCAGGAAATGGGAAGAGAAATCATGCGCATGGAATCTCAA<br>GACCCTGAAAAACGGAGTAGATTGTTCAACCCTCTAGAAGCCATTGATGTTCTACAAGGAAATAAGGTCAGTAAATTTCATTATCTTTATCAT<br>GGTTATACTTCTTCTTCTTCTTCGTTTTTCATTTACTAGTTTTTGGGTACGCACATCACGAGTGTAGTTGGGTGTACCAATTACTATAAACTTAT<br>AGTTTATGACAATCGTGCTCCAAATAAGACTTTAGACATCTTATTATAATG	2,556,562-<br>2,558,628	2,067	1,820
**STRG1650**	AAATTGCAGGGTTCTAAAAAAGTAGAAATATTGGTGGTAGAACGACAAGCATTAAAGGGTGTGAAGCTAAGCACCGAAGCATTTCGGAAAA<br>TGATAAATCTTAGGGTGCTTAAAATCGACGACTTACATATTAGTGGAGATTTTGAGCTATTGTCCAAGGAGCTCAGATGGCTGTCTTGGGAAA<br>GATGTCCTTTAAAATGTATACCATCAAAATTTCCATCTGAGAAACTTGTATTTCTGAATATGAAAGGGAGCAATATCCAAGAATTTGGTTTGA<br>ATTTGCAGTATTGTAGAAATTTGAAAAAGCTGGATCTCTCTGATTGCAAGCGCCTCAGAAGAACTCCAAACTTCAAGGGTTCAAGAAGTCTCG<br>TGGGTTTGGTGCTTGATAATTGCTCAAGTCTGAAGGAGATCCATCCATCAATAGGAAATTTGGACAGACTAATTCATCTTTACCTGAAAGGTT<br>GCGAAAAGATTACGGATCTTACGAGCGGCATATGCCAGCTAAAATCCCTTCAATACTTGGACATTGATGGCTGCTCATCTTTACAAACACTGC<br>CAGTTGATATTGGAGATATGCAAAGACTAAGATCTCTTTCAGCAACTGGAACAGGTATAAGAGAATTGCCTGGATCTGTTGAAATGCTAGGA<br>AATCTTATAGCTTTGGAAATAGGAGGTTCATACTTAGAGGCCAAAAGGAGGTTTTCTCAAACAAGAGTACGCCCCGTAGTATCCTTGTCAAAA<br>TTTATTTTGATTTTACGCCTTCCTTACTGTGGTTTCTCGGAGGGTGATGTTCCTATTAGGAATATTGAGAGTTTATCCAACTTACGTGAATTAGA<br>TTTGAGCGGCAACAATTTCCTCTATCTACCCTTTGATTTTTCCAAGTTATCATTGTTGAGGTGCTTGTATTTGAATGATTGTGAGAACCTTCAAA<br>CACTCCCGTCACTATCAAATTTAGAGTACCTTCATACTCTTGAACTTTGGAATTGCCAAAAACTGGTCAATATTACAGGGTTGGACAACCTCC<br>CTTCAATAGAGAAGATGGAAATGAGAAATTGTACTTGTCTGCAGAATCCAATCAATGAAGGTTTCTTTAGTGCCCATGCTCTATCAATTTCAT<br>CTAGAAAACATCCAATGTATGAGGTTAGTCCCTCTCCCTCTCTGATCTCTCTCTCTCGTATCACATTAATAATTAATGATCTAATTGTGTCTTAA<br>TTTGTCTGATGAAGCCATTACAAATTAATCTCGAAAGCAATGAAATTCTAGATTGGTGCAGCAATAAAGTAACAGCTCCATCGATCTGTTTGA<br>CTATGCCCACAGTACATAATAACAACTTCTTAGGAATGGTTCTCTGGTTTGTTTGCGACCATTGCAATGCACAGGAGCTTGAACACTTCGTTGT<br>TACTGTTGCCCATATAAAGCGTTCATGTTTACTGTGTAATGGGCATTTTCATAGATCAAGTCACGAAGTATCATGTGTATATTACTTATCTTCC<br>GCAAATGATGAACCTTTTAAAGGCCTGAACATCAAAGGCGGGGAACAGATAACAGTAGAGGATGGCAGTGACGGAGGCATAGCAAAGAAG<br>ATAGGGATCCATTTGTTATACTTGGACCAACATGGTAATGTTACATCATTGCCGGGAGTTGTGGATCATTCTTATACTCCCTCCTACCCACAAA<br>GACTTTCAGCAGGGCATATCAACTCAAACAACGACAACATATCCAATGAAATCCTACAAGTGAGGTCCGTATCAGTGGATATCAACAAGCAA<br>AGTTCTGAGAGTGTGTTCGGTACCATGGAAAATGTGTTCCATAGAAATAGGTCATGGACCTGGATCTACCAAATGATAACAATGCCTCTAAA<br>ATGTCTTTTTGGAAACTGTTTCATTATCTTTATCTTGGTTATACATATTCTTC	2,565,200-<br>2,567,202	2,003	1,914
